# Supplementary material for: Stable high-density and maternally inherited Wolbachia infections in Anopheles moucheti and Anopheles demeilloni mosquitoes
Source: Curr Biol. 2021 Jun 7;31(11):2310–2320.e5. doi: 10.1016/j.cub.2021.03.056 (PMC8210651; doi:10.1016/j.cub.2021.03.056)
Supplement: Document S1. Figures S1–S4 and Tables S1–S5, S7, S10, and S11 [file mmc1.pdf]

Current Biology, Volume *31*

Supplemental Information

Stable high-density and maternally inherited *Wolbachia* infections in *Anopheles moucheti* and *Anopheles demeilloni* mosquitoes

Thomas Walker, Shannon Quek, Claire L. Jeffries, Janvier Bandibabone, Vishaal Dhokiya, Roland Bamou, Mojca Kristan, Louisa A. Messenger, Alexandra Gidley, Emily A. Hornett, Enyia R. Anderson, Cintia Cansado-Utrilla, Shivanand Hegde, Chimanuka Bantuzeko, Jennifer C. Stevenson, Neil F. Lobo, Simon C. Wagstaff, Christophe Antonio Nkondjio, Seth R. Irish, Eva Heinz, and Grant L. Hughes

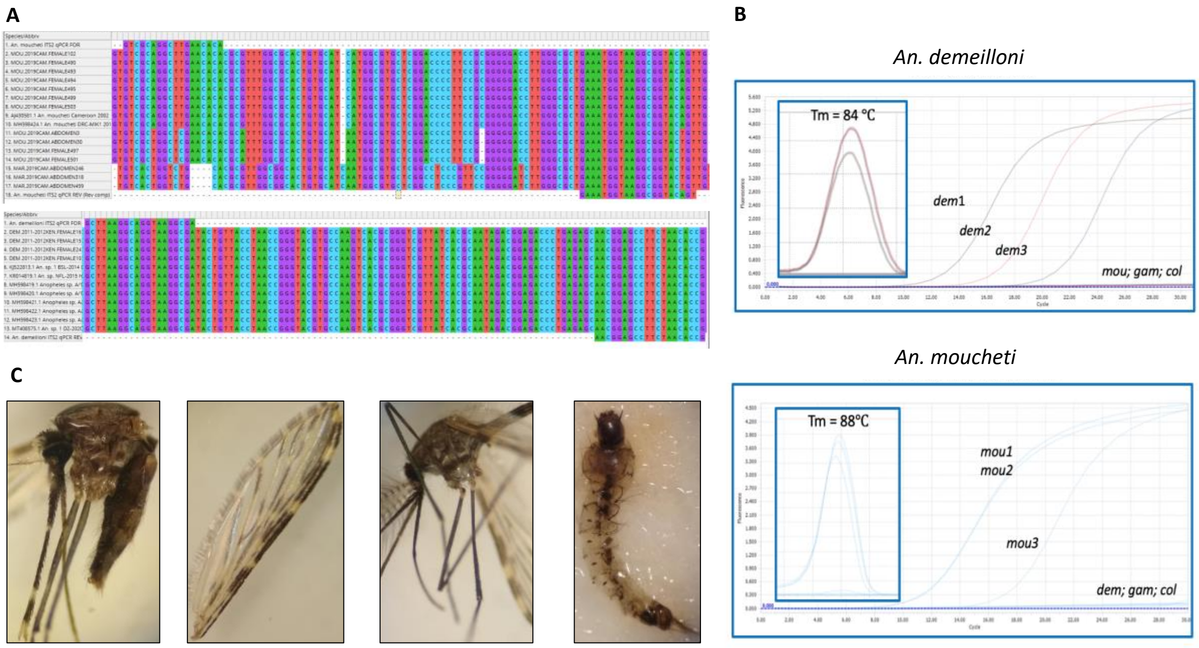

Figure S1: Mosquito identification using qPCR and morphological features. Related to Figure 1.
(A) Alignment of ITS2 sequences and location of species-specific primers.
(B) ITS2 species-specific qPCR fluorescence targeting *An. demeilloni* and *An. moucheti*. Inset = dissociation curves to ensure the correct target sequence was being amplified. dem = *An. demeilloni*, *mou* = *An. moucheti*, *gam* = *An. gambiae* s.s., *col* = *An. coluzzii*.
(C) Images of '*An. species* A'. From left to right: adult female, wing of adult female, adult male and larvae. Independent morphological identification by three individuals using keys confirmed this species is *An. demeilloni*.

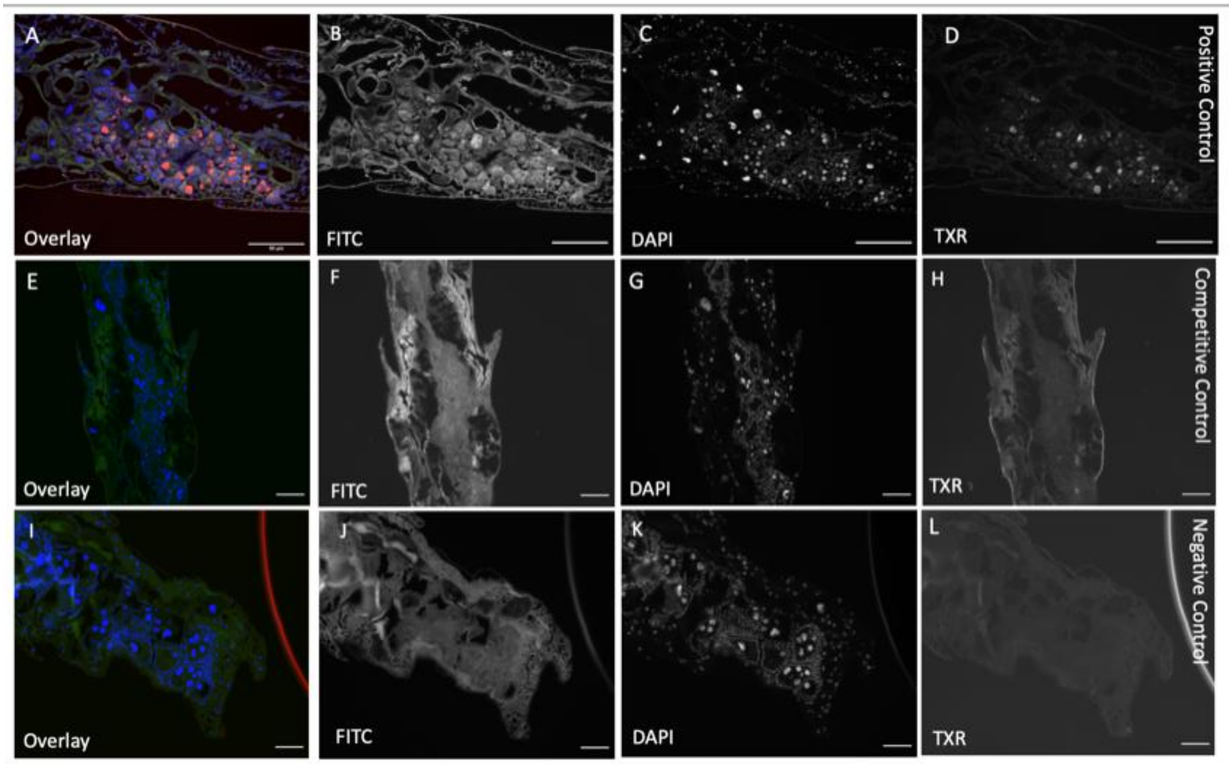

Figure S2: Controls for FISH. Related to Figure 2. Scale bars 90μM.
(A-D) *Wolbachia*-infected *Cx. quinquefasciatus* samples used as a positive control
(E-H) *An. moucheti* competitive control
(I-L) *An. moucheti* no probe control

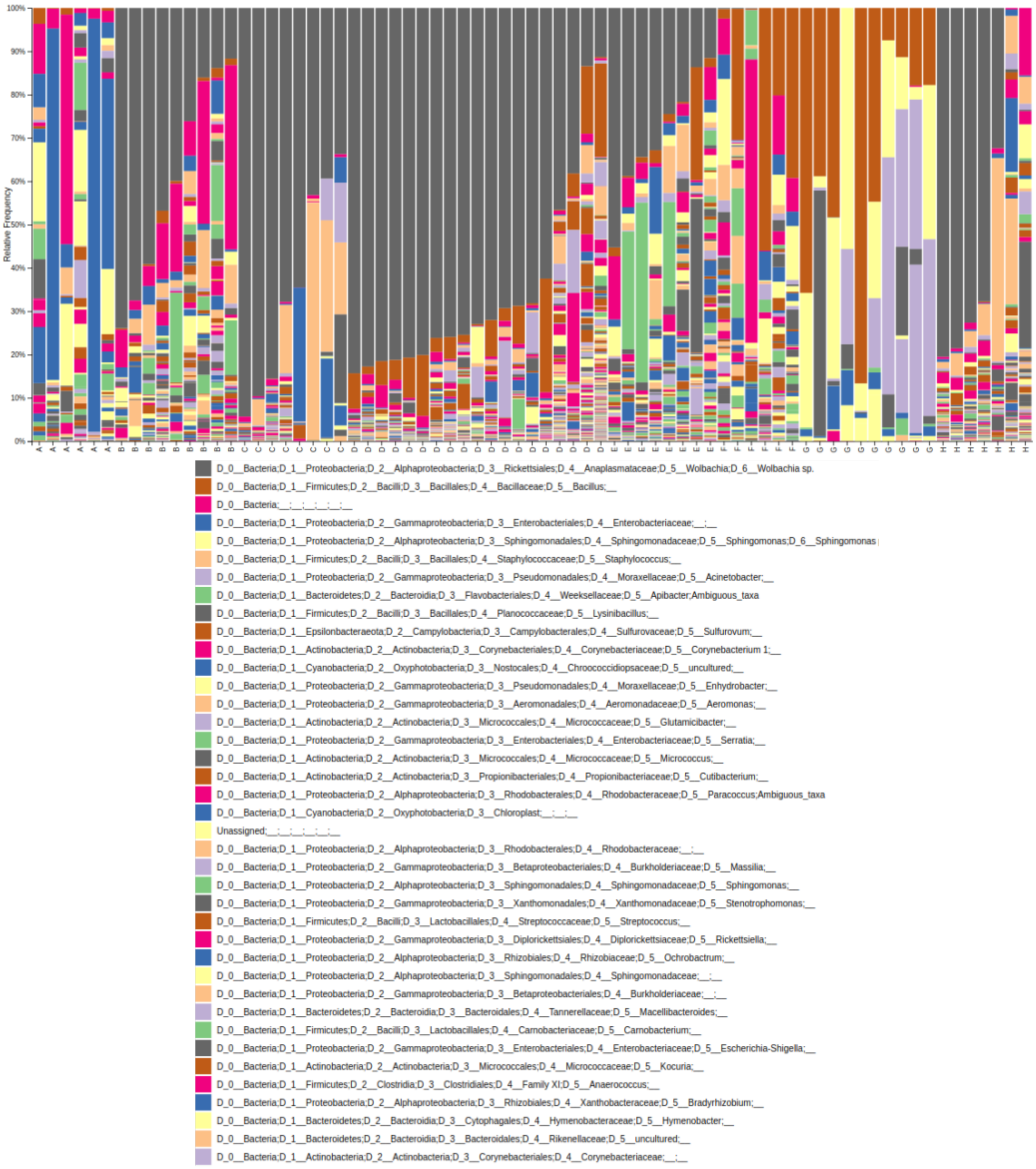

Group ID	Species (body part/generation)	Country (year)	*Wolbachia*+/total
A	*An. demeilloni* (whole/G0)	DRC (2015)	0/6
B	*An. demeilloni* (whole/G0)	DRC (2015)	9/9
C	*An. demeilloni* (whole/G0)	DRC (2019)	8/8
D	*An. moucheti* (abdomen/G0)	Cameroon (2019)	19/19
E	*An. moucheti* (head-thorax/G0)	Cameroon (2019)	8/8
F	*An. moucheti* (head-thorax/G0)	Cameroon (2019)	0/6
G	*An. moucheti* (whole/G0)	Cameroon (2019)	0/10
H	*An. moucheti* (whole/F1)	Cameroon (2019)	5/7

Figure S3: Microbiome relative taxonomic abundance barplots. Related to Figure 3. The relative taxonomic abundance barplots for each sample, as visualised using the qiime taxa barplot command within QIIME2. Sample groups with metadata are as detailed in the table and the legend details the level 7 classification of the 20 most abundant ASVs across all samples. Samples are arranged by group, then by descending % *Wolbachia*. The overwhelming dominance of *Wolbachia* within the microbiome of *An. demeilloni* and *An. moucheti* samples in the *Wolbachia* positive groups can be seen. In addition, the presence of high numbers of *Wolbachia* reads across different years for *An. demeilloni* (groups B and C), and in both the abdomen and head-thorax in *An. moucheti* (groups C and D) is shown. The maternal transmission of *Wolbachia* is demonstrated in the F1 *An. moucheti Wolbachia* positive samples within group H. The diversity of microbes present in these mosquitoes when *Wolbachia* is absent can also been seen (groups A, F, G and H).

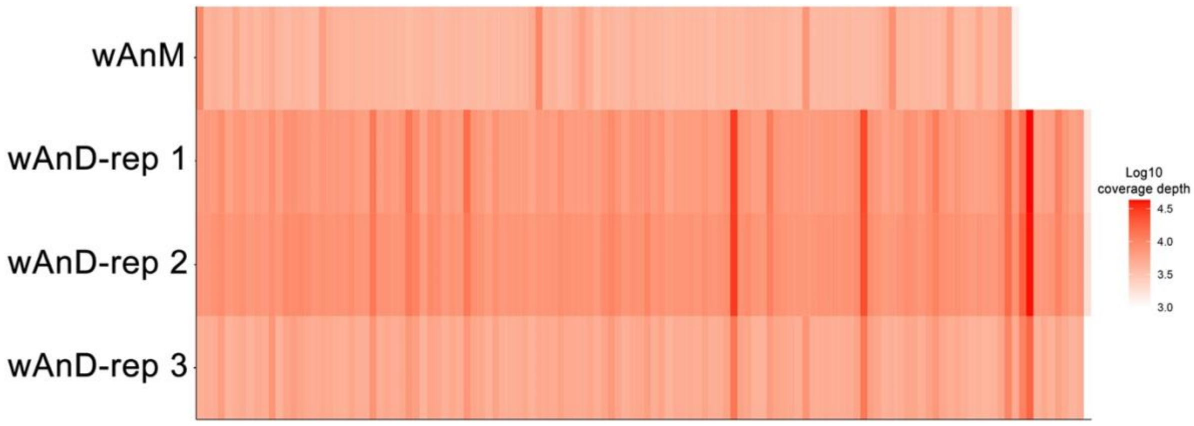

Figure S4. Heatmap representing depth of coverage for the assembled *w*AnM and *w*AnD genomes within 10kbp windows. Related to Figure 5.
Contigs for both genomes were first concatenated into one long assembly, before being separated into 10kbp-long windows. Sequencing data from individual samples were then mapped against the genome, and sequencing depth for each 10kbp window then calculated. Each row represents a single sample that has been aligned to one of the two genomes *w*AnM or *w*AnD, with intensity of red indicating the depth of sequencing as shown by the key to the right.

Sample ID	Location	Year	Species	Gene fragment	GenBank Accession Number
MOU.2015DRC.ABDOMEN7	Lwiro, DRC	2015	*An. moucheti moucheti*	Mosquito host COII	MW250655
MOU.2015DRC.ABDOMEN8	Lwiro, DRC	2015	*An. moucheti moucheti*	Mosquito host COII	MW250656
MOU.2019CAM.FEMALE30	Olama Village, CAM	2019	*An. moucheti moucheti*	Mosquito host COII	MW250657
MOU.2019CAM.FEMALE102	Olama Village, CAM	2019	*An. moucheti moucheti*	Mosquito host COII	MW250658
MOU.2019CAM.FEMALE490	Olama Village, CAM	2019	*An. moucheti moucheti*	Mosquito host COII	MW250659
MOU.2019CAM.FEMALE491	Olama Village, CAM	2019	*An. moucheti moucheti*	Mosquito host COII	MW250660
MOU.2019CAM.FEMALE493	Olama Village, CAM	2019	*An. moucheti moucheti*	Mosquito host COII	MW250661
MOU.2019CAM.FEMALE494	Olama Village, CAM	2019	*An. moucheti moucheti*	Mosquito host COII	MW250662
MOU.2019CAM.FEMALE495	Olama Village, CAM	2019	*An. moucheti moucheti*	Mosquito host COII	MW250663
MOU.2019CAM.FEMALE497	Olama Village, CAM	2019	*An. moucheti cf. moucheti*	Mosquito host COII	MW250664
MOU.2019CAM.FEMALE499	Olama Village, CAM	2019	*An. moucheti moucheti*	Mosquito host COII	MW250665
MOU.2019CAM.FEMALE500	Olama Village, CAM	2019	*An. moucheti moucheti*	Mosquito host COII	MW250666
MOU.2019CAM.FEMALE501	Olama Village, CAM	2019	*An. moucheti cf. moucheti*	Mosquito host COII	MW250667
MOU.2019CAM.FEMALE503	Olama Village, CAM	2019	*An. moucheti moucheti*	Mosquito host COII	MW250668
MOU.2019CAM.ABDOMEN1	Olama Village, CAM	2019	*An. moucheti moucheti*	Mosquito host COII	MW250669
MOU.2019CAM.ABDOMEN2	Olama Village, CAM	2019	*An. moucheti moucheti*	Mosquito host COII	MW250670
MOU.2019CAM.ABDOMEN3	Olama Village, CAM	2019	*An. moucheti cf. moucheti*	Mosquito host COII	MW250671
MOU.2019CAM.ABDOMEN6	Olama Village, CAM	2019	*An. moucheti moucheti*	Mosquito host COII	MW250672
MOU.2019CAM.ABDOMEN7	Olama Village, CAM	2019	*An. moucheti moucheti*	Mosquito host COII	MW250673
MOU.2019CAM.ABDOMEN9	Olama Village, CAM	2019	*An. moucheti moucheti*	Mosquito host COII	MW250674
MOU.2019CAM.ABDOMEN10	Olama Village, CAM	2019	*An. moucheti moucheti*	Mosquito host COII	MW250675
MOU.2019CAM.ABDOMEN11	Olama Village, CAM	2019	*An. moucheti moucheti*	Mosquito host COII	MW250676
MOU.2019CAM.ABDOMEN12	Olama Village, CAM	2019	*An. moucheti moucheti*	Mosquito host COII	MW250677
MOU.2019CAM.ABDOMEN13	Olama Village, CAM	2019	*An. moucheti moucheti*	Mosquito host COII	MW250678
MOU.2019CAM.ABDOMEN15	Olama Village, CAM	2019	*An. moucheti moucheti*	Mosquito host COII	MW250679
MOU.2019CAM.ABDOMEN17	Olama Village, CAM	2019	*An. moucheti moucheti*	Mosquito host COII	MW250680
MOU.2019CAM.ABDOMEN18	Olama Village, CAM	2019	*An. moucheti moucheti*	Mosquito host COII	MW250681
MOU.2019CAM.ABDOMEN19	Olama Village, CAM	2019	*An. moucheti moucheti*	Mosquito host COII	MW250682
MOU.2019CAM.ABDOMEN20	Olama Village, CAM	2019	*An. moucheti moucheti*	Mosquito host COII	MW250683
MOU.2019CAM.ABDOMEN21	Olama Village, CAM	2019	*An. moucheti moucheti*	Mosquito host COII	MW250684
MOU.2019CAM.ABDOMEN30	Olama Village, CAM	2019	*An. moucheti cf. moucheti*	Mosquito host COII	MW250685
MAR.2019CAM.ABDOMEN246	Olama Village, CAM	2019	*An. marshallii*	Mosquito host COII	MW250686
MAR.2019CAM.ABDOMEN318	Olama Village, CAM	2019	*An. marshallii*	Mosquito host COII	MW250687
MAR.2019CAM.ABDOMEN459	Olama Village, CAM	2019	*An. marshallii*	Mosquito host COII	MW250688
DEM.2019DRC.FEMALE3	Lwiro, DRC	2019	*An. demeilloni*	Mosquito host COII	MW250689
DEM.2011-2012KEN.FEMALE16	Western Highlands, KEN	2011-2012	*An. demeilloni*	Mosquito host COII	MW250690
DEM.2011-2012KEN.FEMALE15	Western Highlands, KEN	2011-2012	*An. demeilloni*	Mosquito host COII	MW250691
DEM.2011-2012KEN.FEMALE24	Western Highlands, KEN	2011-2012	*An. demeilloni*	Mosquito host COII	MW250692
DEM.2011-2012KEN.FEMALE10	Western Highlands, KEN	2011-2012	*An. demeilloni*	Mosquito host COII	MW250693
MOU.2019CAM.FEMALE102	Olama Village, CAM	2019	*An. moucheti moucheti*	Mosquito host ITS2	MW257124
MOU.2019CAM.FEMALE490	Olama Village, CAM	2019	*An. moucheti moucheti*	Mosquito host ITS2	MW257125
MOU.2019CAM.FEMALE491	Olama Village, CAM	2019	*An. moucheti moucheti*	Mosquito host ITS2	MW257126
MOU.2019CAM.FEMALE493	Olama Village, CAM	2019	*An. moucheti moucheti*	Mosquito host ITS2	MW257127
MOU.2019CAM.FEMALE494	Olama Village, CAM	2019	*An. moucheti moucheti*	Mosquito host ITS2	MW257128
MOU.2019CAM.FEMALE495	Olama Village, CAM	2019	*An. moucheti moucheti*	Mosquito host ITS2	MW257129
MOU.2019CAM.FEMALE499	Olama Village, CAM	2019	*An. moucheti moucheti*	Mosquito host ITS2	MW257130
MOU.2019CAM.FEMALE500	Olama Village, CAM	2019	*An. moucheti moucheti*	Mosquito host ITS2	MW257131
MOU.2019CAM.FEMALE503	Olama Village, CAM	2019	*An. moucheti moucheti*	Mosquito host ITS2	MW257132
MOU.2019CAM.FEMALE497	Olama Village, CAM	2019	*An. moucheti cf. moucheti*	Mosquito host ITS2	MW257133
MOU.2019CAM.FEMALE501	Olama Village, CAM	2019	*An. moucheti cf. moucheti*	Mosquito host ITS2	MW257134
MOU.2019CAM.ABDOMEN3	Olama Village, CAM	2019	*An. moucheti cf. moucheti*	Mosquito host ITS2	MW257135
MOU.2019CAM.ABDOMEN30	Olama Village, CAM	2019	*An. moucheti cf. moucheti*	Mosquito host ITS2	MW257136
MAR.2019CAM.ABDOMEN246	Olama Village, CAM	2019	*An. marshallii*	Mosquito host ITS2	MW257137
MAR.2019CAM.ABDOMEN318	Olama Village, CAM	2019	*An. marshallii*	Mosquito host ITS2	MW257138
MAR.2019CAM.ABDOMEN459	Olama Village, CAM	2019	*An. marshallii*	Mosquito host ITS2	MW257139
MOU.2015DRC.ABDOMEN1	Lwiro, DRC	2015	*An. moucheti moucheti*	Mosquito host ITS2	MW257140
MOU.2015DRC.ABDOMEN2	Lwiro, DRC	2015	*An. moucheti moucheti*	Mosquito host ITS2	MW257141
MOU.2015DRC.ABDOMEN3	Lwiro, DRC	2015	*An. moucheti moucheti*	Mosquito host ITS2	MW257142

MOU.2015DRC.ABDOMEN4	Lwiro, DRC	2015	*An. moucheti moucheti*	Mosquito host ITS2	MW257143
MOU.2015DRC.ABDOMEN5	Lwiro, DRC	2015	*An. moucheti moucheti*	Mosquito host ITS2	MW257144
MOU.2015DRC.ABDOMEN6	Lwiro, DRC	2015	*An. moucheti moucheti*	Mosquito host ITS2	MW257145
MOU.2015DRC.ABDOMEN7	Lwiro, DRC	2015	*An. moucheti moucheti*	Mosquito host ITS2	MW257146
MOU.2015DRC.ABDOMEN8	Lwiro, DRC	2015	*An. moucheti moucheti*	Mosquito host ITS2	MW257147
2011-2012.KEN.FEMALE16	Western Highlands, KEN	2011-2012	*An. demeilloni*	Mosquito host ITS2	MW257148
2011-2012.KEN.FEMALE15	Western Highlands, KEN	2011-2012	*An. demeilloni*	Mosquito host ITS2	MW257149
2011-2012.KEN.FEMALE24	Western Highlands, KEN	2011-2012	*An. demeilloni*	Mosquito host ITS2	MW257150
2011-2012.KEN.FEMALE10	Western Highlands, KEN	2011-2012	*An. demeilloni*	Mosquito host ITS2	MW257151
MOU.2015DRC.ABDOMEN3 wsp(1)	Lwiro, DRC	2015	*An. moucheti moucheti*	Wolbachia wsp	MW250694
MOU.2019CAM.ABDOMEN1	Olama Village, CAM	2019	*An. moucheti moucheti*	Wolbachia wsp	MW250695
MOU.2019CAM.ABDOMEN2	Olama Village, CAM	2019	*An. moucheti moucheti*	Wolbachia wsp	MW250696
MOU.2019CAM.ABDOMEN3 wsp(2)	Olama Village, CAM	2019	*An. moucheti cf. moucheti*	Wolbachia wsp	MW250697
MOU.2019CAM.ABDOMEN6 wsp(1)	Olama Village, CAM	2019	*An. moucheti moucheti*	Wolbachia wsp	MW250698
MOU.2019CAM.ABDOMEN7	Olama Village, CAM	2019	*An. moucheti moucheti*	Wolbachia wsp	MW250699
MOU.2019CAM.ABDOMEN8	Olama Village, CAM	2019	*An. moucheti*	Wolbachia wsp	MW250700
MOU.2019CAM.ABDOMEN9	Olama Village, CAM	2019	*An. moucheti moucheti*	Wolbachia wsp	MW250701
MOU.2019CAM.ABDOMEN10	Olama Village, CAM	2019	*An. moucheti moucheti*	Wolbachia wsp	MW250702
MOU.2019CAM.ABDOMEN11	Olama Village, CAM	2019	*An. moucheti moucheti*	Wolbachia wsp	MW250703
MOU.2019CAM.ABDOMEN12	Olama Village, CAM	2019	*An. moucheti moucheti*	Wolbachia wsp	MW250704
MOU.2019CAM.ABDOMEN13	Olama Village, CAM	2019	*An. moucheti moucheti*	Wolbachia wsp	MW250705
MOU.2019CAM.ABDOMEN15	Olama Village, CAM	2019	*An. moucheti moucheti*	Wolbachia wsp	MW250706
MOU.2019CAM.ABDOMEN16	Olama Village, CAM	2019	*An. moucheti*	Wolbachia wsp	MW250707
MOU.2019CAM.ABDOMEN17	Olama Village, CAM	2019	*An. moucheti moucheti*	Wolbachia wsp	MW250708
MOU.2019CAM.ABDOMEN18	Olama Village, CAM	2019	*An. moucheti moucheti*	Wolbachia wsp	MW250709
MOU.2019CAM.ABDOMEN19	Olama Village, CAM	2019	*An. moucheti moucheti*	Wolbachia wsp	MW250710
MOU.2019CAM.ABDOMEN20	Olama Village, CAM	2019	*An. moucheti moucheti*	Wolbachia wsp	MW250711
MOU.2019CAM.ABDOMEN30 wsp(2)	Olama Village, CAM	2019	*An. moucheti cf. moucheti*	Wolbachia wsp	MW250712
MOU.2019CAM.FEMALE102 wsp(1)	Olama Village, CAM	2019	*An. moucheti moucheti*	Wolbachia wsp	MW250713
2011-2012.KEN.FEMALE16	Western Highlands, KEN	2011-2012	*An. demeilloni*	Wolbachia wsp	MW250714
2011-2012.KEN.FEMALE15	Western Highlands, KEN	2011-2012	*An. demeilloni*	Wolbachia wsp	MW250715
2011-2012.KEN.FEMALE24	Western Highlands, KEN	2011-2012	*An. demeilloni*	Wolbachia wsp	MW250716
MOU.2019CAM.ABDOMEN3	Olama Village, CAM	2019	*An. moucheti cf. moucheti*	Wolbachia coxA	MW250717
MOU.2019CAM.ABDOMEN30	Olama Village, CAM	2019	*An. moucheti cf. moucheti*	Wolbachia coxA	MW250718
MOU.2019CAM.FEMALE102	Olama Village, CAM	2019	*An. moucheti moucheti*	Wolbachia coxA	MW250719
MOU.2015DRC.ABDOMEN3	Lwiro, DRC	2015	*An. moucheti moucheti*	Wolbachia coxA	MW250720
MOU.2015DRC.ABDOMEN4	Lwiro, DRC	2015	*An. moucheti moucheti*	Wolbachia coxA	MW250721
MOU.2015DRC.ABDOMEN7	Lwiro, DRC	2015	*An. moucheti moucheti*	Wolbachia coxA	MW250722
MOU.2015DRC.ABDOMEN8	Lwiro, DRC	2015	*An. moucheti moucheti*	Wolbachia coxA	MW250723
2011-2012.KEN.FEMALE16 (coxA-1)	Western Highlands, KEN	2011-2012	*An. demeilloni*	Wolbachia coxA	MW250724
2011-2012.KEN.FEMALE15 (coxA-1)	Western Highlands, KEN	2011-2012	*An. demeilloni*	Wolbachia coxA	MW250725
2011-2012.KEN.FEMALE15 (coxA-2)	Western Highlands, KEN	2011-2012	*An. demeilloni*	Wolbachia coxA	MW250726
2011-2012.KEN.FEMALE24 (coxA-1)	Western Highlands, KEN	2011-2012	*An. demeilloni*	Wolbachia coxA	MW250727
MOU.2019CAM.ABDOMEN3	Olama Village, CAM	2019	*An. moucheti cf. moucheti*	Wolbachia gatB	MW250728
MOU.2019CAM.ABDOMEN30	Olama Village, CAM	2019	*An. moucheti cf. moucheti*	Wolbachia gatB	MW250729
MOU.2019CAM.FEMALE102	Olama Village, CAM	2019	*An. moucheti moucheti*	Wolbachia gatB	MW250730
MOU.2015DRC.ABDOMEN3	Lwiro, DRC	2015	*An. moucheti moucheti*	Wolbachia gatB	MW250731
MOU.2015DRC.ABDOMEN4	Lwiro, DRC	2015	*An. moucheti moucheti*	Wolbachia gatB	MW250732
MOU.2015DRC.ABDOMEN7	Lwiro, DRC	2015	*An. moucheti moucheti*	Wolbachia gatB	MW250733
MOU.2015DRC.ABDOMEN8	Lwiro, DRC	2015	*An. moucheti moucheti*	Wolbachia gatB	MW250734
2011-2012.KEN.FEMALE16	Western Highlands, KEN	2011-2012	*An. demeilloni*	Wolbachia gatB	MW250735
2011-2012.KEN.FEMALE15	Western Highlands, KEN	2011-2012	*An. demeilloni*	Wolbachia gatB	MW250736
2011-2012.KEN.FEMALE24	Western Highlands, KEN	2011-2012	*An. demeilloni*	Wolbachia gatB	MW250737
MOU.2019CAM.ABDOMEN3	Olama Village, CAM	2019	*An. moucheti cf. moucheti*	Wolbachia ftsZ	MW250738
MOU.2019CAM.ABDOMEN30	Olama Village, CAM	2019	*An. moucheti cf. moucheti*	Wolbachia ftsZ	MW250739
MOU.2019CAM.FEMALE102	Olama Village, CAM	2019	*An. moucheti moucheti*	Wolbachia ftsZ	MW250740

MOU.2015DRC.ABDOMEN3	Lwiro, DRC	2015	*An. moucheti moucheti*	Wolbachia ftsZ	MW250741
MOU.2015DRC.ABDOMEN4	Lwiro, DRC	2015	*An. moucheti moucheti*	Wolbachia ftsZ	MW250742
MOU.2015DRC.ABDOMEN7	Lwiro, DRC	2015	*An. moucheti moucheti*	Wolbachia ftsZ	MW250743
MOU.2015DRC.ABDOMEN8	Lwiro, DRC	2015	*An. moucheti moucheti*	Wolbachia ftsZ	MW250744
2011-2012.KEN.FEMALE16	Western Highlands, KEN	2011-2012	*An. demeilloni*	Wolbachia ftsZ	MW250745
2011-2012.KEN.FEMALE15	Western Highlands, KEN	2011-2012	*An. demeilloni*	Wolbachia ftsZ	MW250746
2011-2012.KEN.FEMALE24	Western Highlands, KEN	2011-2012	*An. demeilloni*	Wolbachia ftsZ	MW250747
MOU.2019CAM.ABDOMEN3	Olama Village, CAM	2019	*An. moucheti cf. moucheti*	Wolbachia fpbA	MW250748
MOU.2019CAM.ABDOMEN30	Olama Village, CAM	2019	*An. moucheti cf. moucheti*	Wolbachia fpbA	MW250749
MOU.2019CAM.FEMALE102	Olama Village, CAM	2019	*An. moucheti moucheti*	Wolbachia fpbA	MW250750
MOU.2015DRC.ABDOMEN3	Lwiro, DRC	2015	*An. moucheti moucheti*	Wolbachia fpbA	MW250751
MOU.2015DRC.ABDOMEN4	Lwiro, DRC	2015	*An. moucheti moucheti*	Wolbachia fpbA	MW250752
MOU.2015DRC.ABDOMEN7	Lwiro, DRC	2015	*An. moucheti moucheti*	Wolbachia fpbA	MW250753
MOU.2015DRC.ABDOMEN8	Lwiro, DRC	2015	*An. moucheti moucheti*	Wolbachia fpbA	MW250754
2011-2012.KEN.FEMALE16	Western Highlands, KEN	2011-2012	*An. demeilloni*	Wolbachia fpbA	MW250755
2011-2012.KEN.FEMALE15	Western Highlands, KEN	2011-2012	*An. demeilloni*	Wolbachia fpbA	MW250756
2011-2012.KEN.FEMALE24	Western Highlands, KEN	2011-2012	*An. demeilloni*	Wolbachia fpbA	MW250757
MOU.2019CAM.ABDOMEN3	Olama Village, CAM	2019	*An. moucheti cf. moucheti*	Wolbachia hcpA	MW250758
MOU.2019CAM.ABDOMEN30	Olama Village, CAM	2019	*An. moucheti cf. moucheti*	Wolbachia hcpA	MW250759
MOU.2019CAM.FEMALE102	Olama Village, CAM	2019	*An. moucheti moucheti*	Wolbachia hcpA	MW250760
MOU.2015DRC.ABDOMEN3	Lwiro, DRC	2015	*An. moucheti moucheti*	Wolbachia hcpA	MW250761
MOU.2015DRC.ABDOMEN4	Lwiro, DRC	2015	*An. moucheti moucheti*	Wolbachia hcpA	MW250762
MOU.2015DRC.ABDOMEN7	Lwiro, DRC	2015	*An. moucheti moucheti*	Wolbachia hcpA	MW250763
MOU.2015DRC.ABDOMEN8	Lwiro, DRC	2015	*An. moucheti moucheti*	Wolbachia hcpA	MW250764
2011-2012.KEN.FEMALE16	Western Highlands, KEN	2011-2012	*An. demeilloni*	Wolbachia hcpA	MW250765
2011-2012.KEN.FEMALE15	Western Highlands, KEN	2011-2012	*An. demeilloni*	Wolbachia hcpA	MW250766
2011-2012.KEN.FEMALE24	Western Highlands, KEN	2011-2012	*An. demeilloni*	Wolbachia hcpA	MW250767

Table S1: Additional Sanger sequencing sample details for *w*AnM-infected *An. moucheti* and *w*AnD-infected *An. demeilloni* with their associated GenBank accession numbers. Related to STAR Methods.

The location and year of collection, sample codes and the sequenced gene fragment is shown in addition to the GenBank accession number.

species	collection year	collection location	generation	mosquito body part/life cycle stage	Individual extracts analysed	% individuals *Wolbachia+*	mean *16S rRNA* gene copies /ng DNA
An. moucheti	2019	Olama, CAM	G0 (wild)	female whole body	520	44.2% (238/520)	1.48E+05
An. moucheti	2019	Olama, CAM	G0 (wild)	female abdomen	566	66.6% (377/566)	3.36E+05
An. moucheti	2019	Olama, CAM	G0 (wild)	female head-thorax	566	17.5% (99/566)	5.38E+04
An. moucheti	2019	Olama, CAM	F1	larvae	56	23.2% (13/56)	7.70E+03
An. moucheti	2019	Olama, CAM	F1	pupae	8	25.0% (2/8)	7.80E+03
An. moucheti	2019	Olama, CAM	F1	female whole body	7	85.7% (6/7)	2.36E+05
An. moucheti	2019	Olama, CAM	F1	male whole body	5	80.% (4/5)	1.12E+04
An. moucheti	2015	Lwiro, DRC	G0 (wild)	female abdomen	8	75.0% (6/8)	9.05E+03
An. demeilloni	2015	Lwiro, DRC	G0 (wild)	female whole body	178	89.3% (159/178)	6.67E+03
An. demeilloni	2019	Lwiro, DRC	G0 (wild)	female whole body	8	100.0% (8/8)	8.19E+05
An. demeilloni	2019	Lwiro, DRC	F1	eggs (pooled)	1*	100.0% (1/1)	6.99E+04
An. demeilloni	2019	Lwiro, DRC	F1	larvae	11	72.7% (8/11)	3.79E+03
An. demeilloni	2019	Lwiro, DRC	F1	pupae	9	100.0% (9/9)	1.58E+03
An. demeilloni	2019	Lwiro, DRC	F1	male whole body	10	70% (7/10)	2.93E+03
An. demeilloni	2019	Lwiro, DRC	F1	female abdomen	12	91.7% (11/12)	3.55E+04
An. demeilloni	2019	Lwiro, DRC	F1	female head-thorax	13	0.0% (0/13)	0.00E+00
An. demeilloni	2019	Lwiro, DRC	F2	female abdomen	11	90.9% (10/11)	5.76E+05
An. demeilloni	2019	Lwiro, DRC	F2	female head-thorax	11	18.2% (2/11)	5.91E+03
An. demeilloni	2011-2012	Western Highlands, KEN	G0 (wild)	female whole body	302	38.7% (117/302)	2.04E+05

Table S2: *Wolbachia* density of the *w*AnM and *wAnD* strains. Related to Figure 1. Mean *Wolbachia 16S rRNA* gene copies/ng DNA for *Wolbachia*-infected mosquito DNA extracts. *approximately 200 eggs were pooled prior to extraction. CAM = Cameroon, DRC = Democratic Republic of Congo, KEN = Kenya.

Mosquito species	Collection	strain	*WSP* typing allele numbers				
			wsp	HVR1	HVR2	HVR3	HVR4
An. moucheti	Olama, Cameroon	*w*AnM - VAR1	736	10	**296**	10	13
An. moucheti	Olama, Cameroon	*w*AnM - VAR2	**CM 322 (1 diff)**	10	**295**	10	13
An. demeilloni	Lwiro, DRC	*w*AnD	728	254	288	284	23
An. demeilloni	Western Highlands, Kenya	*w*AnD	728	254	288	284	23

Table S3: *w*AnM and *w*AnD *Wolbachia* strain *WSP* typing. Related to Figure 4.
The *wsp* sequence for *w*AnM-VAR2 had 1 nucleotide difference to allele number 322 (CM = closest match) and 3 differences to *w*AnM-VAR1 in hypervariable region (HVR) 2.

**Average X mapping coverage**	*Wolbachia* genomes 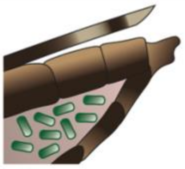		Mosquito genomes 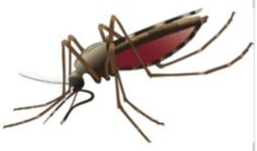	
	min	max	min	max
Unknown *Wolbachia* infection	0.01	2.83	6.29	149.78
An. gambiae (Burkina Faso)[○]	0.03	0.08	149.78	
An. gambiae (DRC)[◇]	0.06	0.11	62.56	
An. coluzzii (Ghana)	0.01	0.03	18.85	
Known *Wolbachia* infection	0.26	148.55	8.19	107.13
An. moucheti	9.40	62.07	34.50	
An. demeilloni	14.96	74.77	22.80	

Table S4: **Average mapping coverage of *Wolbachia* and host mosquito genomes. Related to Figure 5.** Average mapping coverage of *Wolbachia* and host mosquito genomes. The average minimum and maximum coverage are shown comparing *Anopheles* species and arthropods with or without a known *Wolbachia* strain. Due to no genome being available for *An. demeilloni* and *An. moucheti*, the genome of *An. funestus* was used for comparison (see methods).

**Strain identifier**	**Supergroup**	**Genome size**	**GC %**	**Contigs**	**Predicted genes**
*w*AnM	B	1,133,840	33.59%	179	1,164
*w*AnD	B	1,232,500	33.58%	64	1,314

Table S5. General characteristics of the *w*AnD and *w*AnM genomes. Related to Figure 5.

Accession	Short name	Host name	Publish date
GCA_000008025.1	wDmel	Drosophila melanogaster	16/01/2002
GCA_000073005.1	wPip	Culex quinquefasciatus Pel	13/06/2008
GCA_000376585.1	wNo	Drosophila simulans strain No	22/04/2013
GCA_000376605.1	wHa	Drosophila simulans strain Ha	22/04/2013
GCA_000475015.1	wMelPop	Drosophila melanogaster strain popcorn	21/10/2013
GCA_000530755.1	wOv	Onchocerca volvulus	24/01/2014
GCA_000953315.1	wAu	Drosophila simulans strain Au	15/10/2014
GCA_001648015.1	wDacB	Dactylopus coccus supergroup B	19/05/2016
GCA_001648025.1	wDacA	Dactylopus coccus supergroup A	19/05/2016
GCA_001758565.1	wInc_Cu	Drosophila incompta	11/10/2016
GCA_001758585.1	wInc_SM	Drosophila incompta	11/10/2016
GCA_001931755.2	wFcan	Folsoma candida	25/06/2018
GCA_002204235.2	wWb	Wuchereria bancrofti	07/07/2017
GCA_002300525.1	wSpc	Drosophila subpulchrella	13/09/2017
GCA_003344345.1	wCon	Cylisticus convexus	27/07/2018
GCA_003704235.1	wFex	Formica execta	31/10/2018
GCA_003999585.1	wBtab	Bemisia tabaci	08/01/2019
GCA_004795415.1	wAlbB	Aedes albopictus	15/04/2019
GCA_004795935.1	wBm	Brugia malayi	30/08/2019
GCA_004795975.1	wMau	Drosophila mauritania	15/04/2019
GCA_007115015.1	wStri	Laodelphax striatellus	18/07/2019
GCF_000174095.1	wUni	Muscidufax uniraptor	24/03/2009
GCF_000204545.1	wNvit	Nasonia vitripennis	22/04/2011
GCF_000306885.1	wOo	Onchocerca ochengi	30/07/2012
GCF_000331595.1	wDcit	Diaphoria citri	15/01/2013
GCF_000333795.1	wDsuzu	Drosophila suzukii	11/01/2013
GCF_000689175.1	wGmm	Glossina morsitans morsitans	09/05/2014
GCF_000723225.2	wPip_Mol	Culex molestus	20/04/2015
GCF_000829315.1	wClec	Cimex lectularius	10/06/2014
GCF_001027565.1	wAvul	Armadillidium vulgare	18/06/2015
GCF_001266585.1	wOpbru	Operophtera brumata	11/08/2015
GCF_001439985.1	wTPRE	Trichogramma pretiosum	07/01/2016
GCF_001675695.1	wNfla	Nomada flava	23/06/2016
GCF_001675715.1	wNleu	Nomada leucophthalma	23/06/2016
GCF_001675775.1	wNpa	Nomada panzeri	23/06/2016
GCF_001675785.1	wNferru	Nomada ferruginata	23/06/2016
GCF_005862095.1	wSan	Drosophila santomea	27/05/2019
GCF_005862115.1	wYak	Drosophila yakuba	27/05/2019
GCF_005862135.1	wTei	Drosophila teissieri	27/05/2019
GCF_006334525.1	wGBW	Leptopilina clavipes	12/06/2019
GCF_006542295.1	wCauA	Carposina sasakii	02/07/2019
GCF_007115045.1	wLug	Nilaparvata lugens	18/07/2019
GCF_008033215.1	wDana	Drosophila ananassae	20/08/2019
GCF_008245065.1	wMeg	Chrysoma megacephala	03/09/2019
GCF_009012935.1	wOneA1	Nasonia oneida	11/10/2019
wLs 2.0, http://nematodes.org/genomes/litomosoides_sigmodontis/	wLs	Litomosoides sigmodontis	06/08/2012
wDi, http://nematodes.org/genomes/dirofilaria_immitis/	wDi	Dirofilaria immitis	06/08/2012
-	wAnM_draft_genome	Anopheles moucheti	-
-	wAnD_draft_genome	Anopheles species A	-
Supplementary material, DOI 10.7717/peerj.5486	wAnsA_Pascar_genome	Anopheles species A	03/09/2018

Table S7: *Wolbachia* genomes used for comparison. Related to STAR Methods.
Existing *Wolbachia* genomes used in this study for comparison against the assembled genomes.

**Gene target and oligonucleotide primer sequences**	**Reference**
Mosquito ITS2:<br>5'-TGTGAACTTGCAGGACACAT-3'; 5'-TATGCTTAAATTYAGGGGGT-3'	[S1]
Mosquito *COII*:<br>5'-TCTAATATGGGAGATTAGTGC-3'; 5'-ACTTGCTTTCAGTCATCTAATG-3'	[S2]
Wolbachia wsp:<br>5'-TGGTCCAATAAGTGATGAAGAAAC-3'; 5'-AAAAATTAAACGCTACTCCA-3'	[S3]
Wolbachia 16S rRNA:<br>5'-CATACCTATTCGAAGGGATA-3'; 5'-AGCTTCGAGTGAAACCAATTC-3	[S4]
Wolbachia coxA:<br>5'-TTGGRGCRATYAACTTTATAG-3'; 5'-CTAAAGACTTTKACRCCAGT-3'	[S5]
Wolbachia ftsZ:<br>5'-ATYATGGARCATATAAARGATAG-3'; 5'-TCRAGYAATGGATTRGATAT-3'	[S5]
Wolbachia hcpA:<br>5'-GAAATARCAGTTGCTGCAAA-3'; 5'-GAAAGTYRAGCAAGYTCTG-3'	[S5]
Wolbachia fbpA:<br>5'-GCTGCTCCRCTTGGYWTGAT-3'; 5'-CCRCCAGARAAAAYYACTATTC-3	[S5]
Wolbachia gatB:<br>5'-GAKTTAAAYCGYGCAGGBGTT-3'; 5'-TGGYAAYTCRGGYAAAGATGA-3'	[S5]
Wolbachia 16S rRNA (qPCR):<br>5′-CATACCTATTCGAAGGGATAG-3′; 5′-TTGCGGGACTTAACCCAACA-3′	[S6]
An. moucheti ITS2:<br>5'-GTCGCAGGCTTGAACACA-3'; 5'-ACTGTACCGCCTTACCATTTC-3'	In this study
An. demeilloni ITS2<br>5'-GCTTAAGGCAGGTAAGGCGA-3'; 5'-CGGTGTTAGAAGGCTCCGTT-3'	In this study
Bacterial *16S rRNA* V3-V4:<br>5'-CCTACGGGNGGCWGCAG-3'; 5'-GGACTACHVGGGTATCTAATCC-3'	[S7]

Table S10: Primers used in this study. Related to STAR Methods.

**Species**	**number collected**
An. nili	1
An. paludis	26
An. gambiae s.l.	32
An. ziemanni	451
An. marshallii	855
An. moucheti s.l.	2652
Total	**4017**

Table S11. *Anopheles* species collected from Olama Village, Cameroon using human landing catches in 2019. Related to STAR Methods.

Supplemental References

S1. Beebe N.W., Saul A. Discrimination of all members of the Anopheles punctulatus complex by polymerase chain reaction--restriction fragment length polymorphism analysis. Am J Trop Med Hyg. 1995;**53**(5):478-81.

S2. Ndo C., Antonio-Nkondjio C., Cohuet A., Ayala D., Kengne P., Morlais I., Awono-Ambene P.H., Couret D., Ngassam P., Fontenille D., et al. Population genetic structure of the malaria vector Anopheles nili in sub-Saharan Africa. Malar J. 2010;**9**:161.

S3. Zhou W., Rousset F., O'Neil S. Phylogeny and PCR-based classification of Wolbachia strains using wsp gene sequences. Proc Biol Sci. 1998;**265**(1395):509-15.

S4. Werren J.H., Windsor D.M. Wolbachia infection frequencies in insects: evidence of a global equilibrium? Proc Biol Sci. 2000;**267**(1450):1277-85.

S5. Baldo L., Dunning Hotopp J.C., Jolley K.A., Bordenstein S.R., Biber S.A., Choudhury R.R., Hayashi C., Maiden M.C., Tettelin H., Werren J.H. Multilocus sequence typing system for the endosymbiont Wolbachia pipientis. Appl Environ Microbiol. 2006;**72**(11):7098-110.

S6. Gomes F.M., Hixson B.L., Tyner M.D.W., Ramirez J.L., Canepa G.E., Alves E.S.T.L., Molina-Cruz A., Keita M., Kane F., Traore B., et al. Effect of naturally occurring Wolbachia in Anopheles gambiae s.l. mosquitoes from Mali on Plasmodium falciparum malaria transmission. Proc Natl Acad Sci U S A. 2017;**114**(47):12566-71.

S7. Klindworth A., Pruesse E., Schweer T., Peplies J., Quast C., Horn M., Glockner F.O. Evaluation of general 16S ribosomal RNA gene PCR primers for classical and next-generation sequencing-based diversity studies. Nucleic Acids Res. 2013;**41**(1):e1.
