# Supplementary material for: Stable high-density and maternally inherited Wolbachia infections in Anopheles moucheti and Anopheles demeilloni mosquitoes
Source: Curr Biol. 2021 Jun 7;31(11):2310–2320.e5. doi: 10.1016/j.cub.2021.03.056 (PMC8210651; doi:10.1016/j.cub.2021.03.056)
Supplement: Document S2. Article plus supplemental information [file mmc5.pdf]

Current Biology

Article

Stable high-density and maternally inherited *Wolbachia* infections in *Anopheles moucheti* and *Anopheles demeilloni* mosquitoes

Highlights

- High-density *Wolbachia* strains found in *An. moucheti* and *An. demeilloni* mosquitoes
- Infections are visualized in the ovaries, and maternal transmission was observed
- Sequencing at depths and coverages comparable to other known *Wolbachia* strains
- Homologs of cytoplasmic incompatibility factor genes are present in both genomes

Authors

Thomas Walker, Shannon Quek, Claire L. Jeffries, ..., Seth R. Irish, Eva Heinz, Grant L. Hughes

Correspondence

thomas.walker@lshtm.ac.uk (T.W.), grant.hughes@lstmed.ac.uk (G.L.H.)

In brief

Wolbachia is an endosymbiotic bacterium found in a variety of insect species and can prevent pathogen transmission in mosquitoes. Walker et al. provide concrete evidence for high-density *Wolbachia* infections in the malaria vectors *Anopheles moucheti* and *An. demeilloni* and present near-complete genomes of these *Wolbachia* strains.

Walker et al., 2021, Current Biology *31*, 2310–2320
June 7, 2021 © 2021 The Author(s). Published by Elsevier Inc.
https://doi.org/10.1016/j.cub.2021.03.056

CellPress

CellPress
OPEN ACCESS

Current Biology

Article

Stable high-density and maternally inherited *Wolbachia* infections in *Anopheles moucheti* and *Anopheles demeilloni* mosquitoes

Thomas Walker,[1,13,*] Shannon Quek,[2,13] Claire L. Jeffries,[1,13] Janvier Bandibabone,[3] Vishaal Dhokiya,[1] Roland Bamou,[4,5] Mojca Kristan,[1] Louisa A. Messenger,[1] Alexandra Gidley,[1] Emily A. Hornett,[2,6] Enyia R. Anderson,[2] Cintia Cansado-Utrilla,[2] Shivanand Hegde,[2] Chimanuka Bantuzeko,[3] Jennifer C. Stevenson,[7,8] Neil F. Lobo,[9] Simon C. Wagstaff,[10] Christophe Antonio Nkondjio,[4] Seth R. Irish,[11] Eva Heinz,[12] and Grant L. Hughes[2,14,15,*]

[1]Department of Disease Control, Faculty of Infectious and Tropical Diseases, London School of Hygiene and Tropical Medicine, London WC1E 7HT, UK
[2]Departments of Vector Biology and Tropical Disease Biology, Centre for Neglected Tropical Diseases, Liverpool School of Tropical Medicine, Liverpool, UK
[3]Laboratoire d'entomologie médicale et parasitologie, Centre de Recherche en Sciences Naturelles (CRSN/LWIRO), Sud-Kivu, Democratic Republic of Congo
[4]Laboratoire de Recherche sur le Paludisme, Organisation de Coordination pour la lutte contre les Endémies en Afrique Centrale (OCEAC), B.P. 288, Yaoundé, Cameroon
[5]Vector Borne Diseases Laboratory of the Applied Biology and Ecology Research Unit (VBID-URBEA), Department of Animal Biology, Faculty of Science of the University of Dschang, P.O. Box 067, Dschang, Cameroon
[6]Institute of Integrative Biology, University of Liverpool, Liverpool, UK
[7]Macha Research Trust, Choma District, Zambia
[8]Department of Molecular Microbiology and Immunology, Bloomberg School of Public Health, Johns Hopkins University, Baltimore, MD, USA
[9]Eck Institute for Global Health, University of Notre Dame, Notre Dame, IN, USA
[10]Centre for Drugs and Diagnostics, Department of Tropical Disease Biology, Liverpool School of Tropical Medicine, Liverpool, UK
[11]Entomology Branch, Division of Parasitic Diseases and Malaria, Center for Global Health, Centers for Disease Control and Prevention, Atlanta, GA 30033, USA
[12]Departments of Vector Biology and Clinical Sciences, Liverpool School of Tropical Medicine, Liverpool, UK
[13]These authors contributed equally
[14]Twitter: @mosquitomicrobe
[15]Lead contact
*Correspondence: thomas.walker@lshtm.ac.uk (T.W.), grant.hughes@lstmed.ac.uk (G.L.H.)
https://doi.org/10.1016/j.cub.2021.03.056

SUMMARY

Wolbachia, a widespread bacterium that can reduce pathogen transmission in mosquitoes, has recently been reported to be present in *Anopheles* (*An.*) species. In wild populations of the *An. gambiae* complex, the primary vectors of *Plasmodium* malaria in Sub-Saharan Africa, *Wolbachia* DNA sequences at low density and infection frequencies have been detected. As the majority of studies have used highly sensitive nested PCR as the only method of detection, more robust evidence is required to determine whether *Wolbachia* strains are established as endosymbionts in *Anopheles* species. Here, we describe high-density *Wolbachia* infections in geographically diverse populations of *An. moucheti* and *An. demeilloni*. Fluorescent *in situ* hybridization localized a heavy infection in the ovaries of *An. moucheti*, and maternal transmission was observed. Genome sequencing of both *Wolbachia* strains obtained genome depths and coverages comparable to those of other known infections. Notably, homologs of cytoplasmic incompatibility factor (*cif*) genes were present, indicating that these strains possess the capacity to induce the cytoplasmic incompatibility phenotype, which allows *Wolbachia* to spread through host populations. These strains should be further investigated as candidates for use in *Wolbachia* biocontrol strategies in *Anopheles* aiming to reduce the transmission of malaria.

INTRODUCTION

The endosymbiotic bacterium *Wolbachia* is currently being deployed in the field for mosquito population replacement and suppression control strategies to reduce pathogen transmission. These approaches are showing great promise in *Aedes* (*Ae.*) mosquitoes, particularly *Ae. aegypti*,[1–5] which is the main vector of arboviruses such as dengue virus. However, translating this control strategy into *Anopheles* mosquitoes for malaria control is proving more challenging, due to the diversity of malaria vector species and the inability to create stable *Wolbachia* transinfected lines. The development of novel malaria vector control

 © 2021 The Author(s). Published by Elsevier Inc.
This is an open access article under the CC BY license (http://creativecommons.org/licenses/by/4.0/).

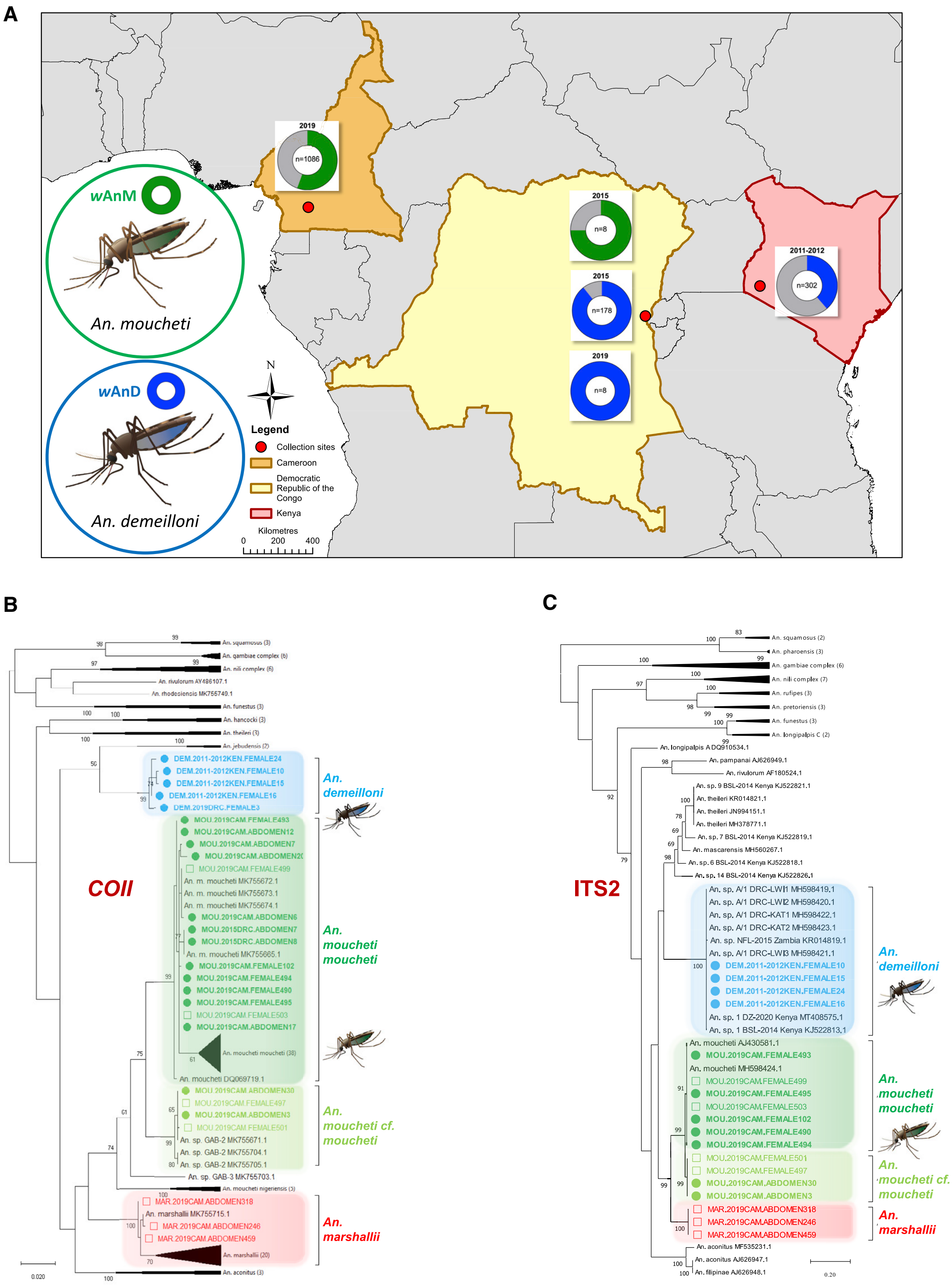

Figure 1. Mosquito collection sites, *Wolbachia* prevalence, and host mosquito phylogenetic analysis

(A) *Wolbachia* prevalence rates in wild adult female mosquitoes for the *w*AnD strain in *An. demeilloni* and *w*AnM strain in *An. moucheti* are denoted in blue and green, respectively.

(B) Mosquito *COII* phylogenetic tree with the highest log likelihood (−4,605.97). The analysis involved 130 nucleotide sequences with a total of 735 positions in the final dataset. Filled circles, *Wolbachia*-infected individuals; open squares, uninfected individuals.

(C) Mosquito ITS2 phylogenetic tree with the highest log likelihood (−11,797.51). The analysis involved 71 nucleotide sequences. There was a total of 1,368 positions in the final dataset. Filled circles, *Wolbachia*-infected individuals; open squares, uninfected individuals.

(legend continued on next page)

tools is highly desirable, as the emergence of insecticide resistance impacts the effectiveness of current control strategies.[6]

Wolbachia can induce two desirable properties in mosquitoes that are exploited for vector control; inhibition of pathogens and cytoplasmic incompatibility (CI), a reproductive phenotype that allows this bacterium to invade host populations. There is growing evidence that *Wolbachia* could be used for malaria biocontrol if stable lines are developed. Transient infections in *An. gambiae*[7] and stable transinfected lines in *An. stephensi*[8] demonstrated a reduction in *Plasmodium* (*P.*) malaria parasites. *Wolbachia* was also able to spread through caged *An. stephensi* populations by CI,[8] although some fitness costs were observed.

Although, for many years, *Anopheles* were thought to be impervious to *Wolbachia* infection,[9,10] several recent reports detect *Wolbachia* DNA in a range of species.[11–18] However, the detection of gene sequences does not confirm the presence of endosymbiotic (or even living) bacteria,[19] given the possibility of environmental contamination or integration into the host genome.[20] The majority of these studies are limited to the amplification of only a few genes (particularly *16S rRNA*), and these findings have been extrapolated to conclude the presence of genuine *Wolbachia* infections. This is problematic, given the high possibility of amplifying prokaryotic *16S rRNA* genes from non-living cells[19] and the detection of *Wolbachia 16S rRNA* sequences in water containers inhabited by mosquito larvae.[20] Furthermore, the prominent use of nested *16S rRNA* PCR to detect low-density strains has led to questions on the validity of concluding that these strains represent stable natural infections,[20,21] and very low prevalence rates in wild mosquito populations suggest that these are unlikely to be CI-inducing strains.

Previously, we identified relatively higher density *Wolbachia* strains in *An. moucheti*, *An. species* A, and an unclassified *Anopheles* species, which represent potentially more favorable candidates for *Wolbachia*-based malaria biocontrol strategies.[18,22] *Anopheles moucheti* is a highly anthropophilic malaria vector found in the forested areas of Western and Central Africa, and there is high genetic diversity in populations,[23] which could influence the prevalence of *Wolbachia* resident strains. *An*. *species* A is a less well-known species found at high altitudes, and its role in malaria transmission is still to be fully determined. Here, we provide robust evidence for these high-density natural *Wolbachia* strains in diverse geographical mosquito populations. These endosymbiotic bacteria can be visualized in the ovaries, are maternally inherited, and dominate the mosquito microbiome. We also report the assembly of near-complete *Wolbachia* genomes and provide evidence that these strains are likely CI inducing from the presence of CI factor (*cif*) gene homologs.

RESULTS

High *Wolbachia* prevalence rates in wild populations

Wolbachia strains that are efficiently maternally transmitted, with high vertical transmission rates combined with induction of CI, can result in invasion of mosquito populations and high prevalence rates. Here, we undertook high-throughput screening examining 1,582 mosquitoes from Cameroon, the Democratic Republic of Congo (DRC), and Kenya to determine both *Wolbachia* prevalence in wild populations and evidence of vertical transmission. *Wolbachia* qPCR analysis of a large number of wild adult female *An. moucheti* from Cameroon (n = 1,086) revealed an overall prevalence of 56.6% for the *w*AnM strain (Figure 1A) which we had previously discovered in the DRC.[18] Host genetic diversity was observed with the presence of two subgroups ("*An. moucheti moucheti*" and "*An. moucheti cf. moucheti*") (Figures 1B, 1C, S1A, and S1B; Table S1). We had previously discovered a novel *Wolbachia* strain in an unidentified *Anopheles* species[18] (originally referred to as *w*AnsA in *An. species* A), which is now confirmed as *An. demeilloni* (Figure S1). The *w*AnD strain in *An. demeilloni* was detected in 38.7% (117/302) of females from Kenya in 2011–2012, 89.3% (159/178) of females from the DRC in 2015, and 100% (n = 8) of females from the DRC in 2019 (Figure 1A).

Evidence that *Wolbachia* strains are likely maternally inherited and can be visualized in mosquito ovaries

The relatively high prevalence rates we found in *An. moucheti* and *An. demeilloni*, compared to those reported for species within the *An. gambiae* complex and *An. funestus*, led us to speculate that vertical transmission was maintaining *Wolbachia* in these populations at high rates. We detected *w*AnM in the resulting F1 generation from wild-caught *An. moucheti* females from Cameroon and *w*AnD in the F1 and F2 *An. demeilloni* generations resulting from wild-caught females from the DRC in all developmental stages (Table S2). However, difficulties maintaining colonies beyond early generations prevented a more comprehensive assessment of maternal transmission efficiency. Several recent studies have called for microscopy to validate PCR data when determining the presence of *Wolbachia* strains in wild mosquito populations.[20,21] As such, we undertook fluorescent *in situ* hybridization (FISH) to visualize *Wolbachia* in the ovaries of wild-caught *An. moucheti*, and a heavy infection was observed in the ovarian egg chambers (Figures 2 and S2). *Wolbachia* could clearly be seen in the oocyte surrounding the nuclei. Some ovarian follicles had a high density *w*AnM infection, while for others, the infection was sparse, which may explain the heterogenous infection prevalence in field populations.

Wolbachia strains are high density and infect somatic tissues

Most studies that have identified *Wolbachia* in *Anopheles* species have used nested PCR, indicating low-density infections. Here, we used qPCR on large cohorts of wild-caught females and showed significant variation in *Wolbachia* density across mosquito species, body parts, and life cycle stages (Table S2). When comparing the density of *w*AnM in all *Wolbachia*-infected abdomens (n = 377) and all *Wolbachia*-infected head-thorax extractions (n = 99) from *An. moucheti* wild-caught females from Cameroon, the density was significantly higher in abdomen extractions, $t(480) = 4.538$, $p < 0.0001$ (Figure 3A). As expected, the density in the abdomen was also significantly higher than

In (B) and (C), reference numbers of additional sequences obtained from GenBank (accession numbers) are shown unless the subtree is compressed. The trees are drawn to scale, with branch lengths measured in the number of substitutions per site.
See also Figure S1 and Table S1.

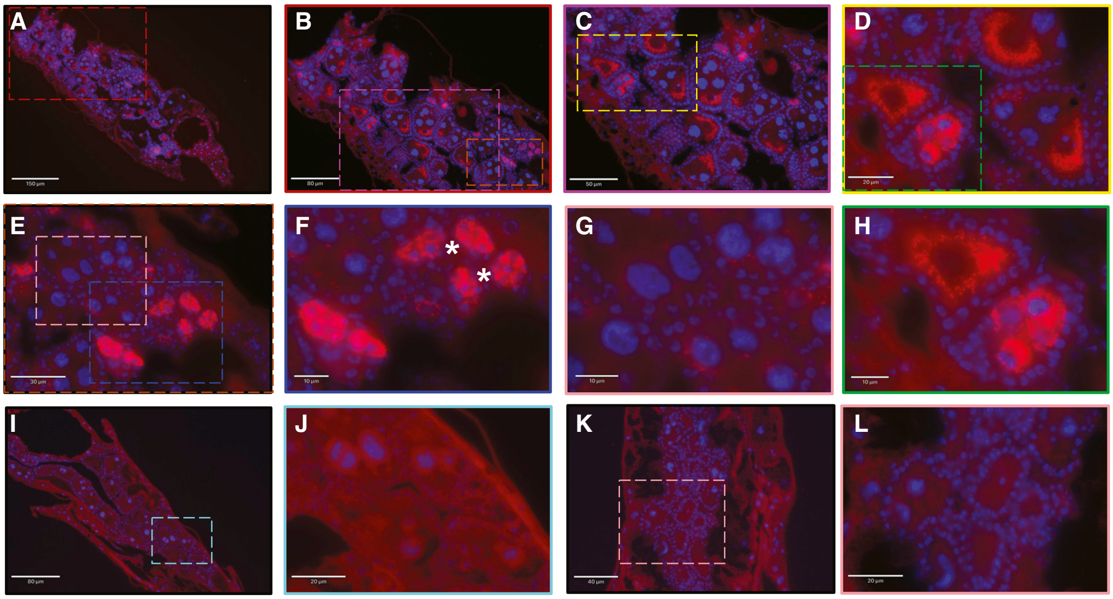

Figure 2. FISH *Wolbachia* visualization in the ovaries

Wolbachia was primarily located in the ovarian follicles (A–H). Colored boxes indicate area of magnification for subsequent images. Within the same ovary, some ovarian follicles are sparsely infected with *Wolbachia* (E and magnification in G), while others have a heavy infection (C, D, and H; E and F). Asterisks indicate infection in the secondary follicles. *Wolbachia* was imaged with an Alexa 590-labeled probe targeting the *Wolbachia 16S rRNA* gene (red), and DNA was stained with DAPI (blue). No probe control images (I–L) show no fluorescent signal (images in I and J and in K and L are for two separate individuals). FISH analysis revealed that 9/16 individuals were *Wolbachia* infected.
See also Figure S2.

in the corresponding head-thorax samples from the same individuals, $t(91) = 7.17$, $p < 0.0001$ (paired t test). Interestingly, we found a significantly higher *w*AnD density in *An. demeilloni* whole adult females collected from Kenya in 2011–2012 (n = 117) compared with those from the DRC in 2015 (n = 158), $t(293) = 12.79$, $p < 0.0001$ (Figure 3A). Although *An. demeilloni* is found at high altitudes in both countries, there are other environmental factors, such as temperature, that can influence *Wolbachia* density in wild mosquito populations. When comparing the overall *Wolbachia* densities between strains, the *w*AnM strain in *An. moucheti* from Cameroon collected in 2019 (n = 238) was significantly higher compared to the *w*AnD strain in *An. demeilloni* from both the DRC in 2015 (n = 158), $t(394) = 7.05$, $p < 0.0001$; and Kenya in 2011–2012 (n = 117), $t(353) = 2.10$, $p = 0.037$.

Wolbachia strains dominate the microbiome

To further confirm high-density strains, we analyzed the composition of bacterial species to determine the relationship of resident *w*AnD and *w*AnM strains and other bacteria (Figures 3B and S3). For *An. demeilloni* females collected from the DRC in 2015 (n = 9), *Wolbachia* was the dominant amplicon sequence variant (ASV) when present, comprising an average 38.1% of total *16S rRNA* reads. In *An. demeilloni* females collected in 2019 (n = 8), *Wolbachia* reads comprised an average of 72.6% of the microbiome. For comparison, we analyzed a selection of *An. demeilloni* 2015 wild-caught females that were *Wolbachia* negative by qPCR (n = 6) and found no *Wolbachia* reads (Figure S3). For *An. moucheti*, *Wolbachia* was the dominant ASV in abdomens (average, 59.2%, n = 19) and in head-thorax samples (average, 29.7%, n = 8) when present (Figure 3B). Our microbiome data corroborate our PCR results with minimal *Wolbachia* reads in our uninfected *An. moucheti* head-thorax samples (n = 6).

Wolbachia strains show consistent allelic profiles across countries

Another characteristic of stably infected *Wolbachia* strains is the presence of the same strain in geographically distinct populations of the same insect species. We found identical multilocus strain typing (MLST) allelic profiles for *w*AnM-infected *An. moucheti* (n = 3) from Cameroon in comparison to those from the DRC.[18] Further analysis of the *Wolbachia* surface protein (*wsp*) gene (n = 49) resulted in two specimens with the same three SNPs (Figure 4A) seen within hypervariable region 2 (Table S3). Using mosquito *COII* gene and ITS2 region phylogeny, we found that the two variant *w*AnM *Wolbachia wsp* sequences were from *An. m. cf. moucheti*, whereas the non-variants (n = 47) were from *An. m. moucheti* (Figures 1B and 1C). No *wsp* gene sequence variation was observed when comparing *w*AnD-infected *An. demeilloni* from Kenya (n = 29) to that from the DRC (Figure 4A; Table S3). Identical MLST sequences and allelic profiles were seen for *w*AnD-infected *An. demeilloni* from Kenya (n = 3) compared to those from the DRC,[18] and similar *coxA* gene sequence variants (3 SNPs) were also found (Figure 4B).[18]

Wolbachia genome sequencing depths

Whole-genome sequencing was undertaken for *An. demeilloni* (*w*AnD) and *An. moucheti* (*w*AnM), in addition to *An. coluzzii* (*w*Anga-Ghana) and five *An. gambiae* s.s. from the DRC that were *Wolbachia* positive by PCR[18] (Figures 5 and S4; Tables S4 and S5). We compared the genome coverage depths against

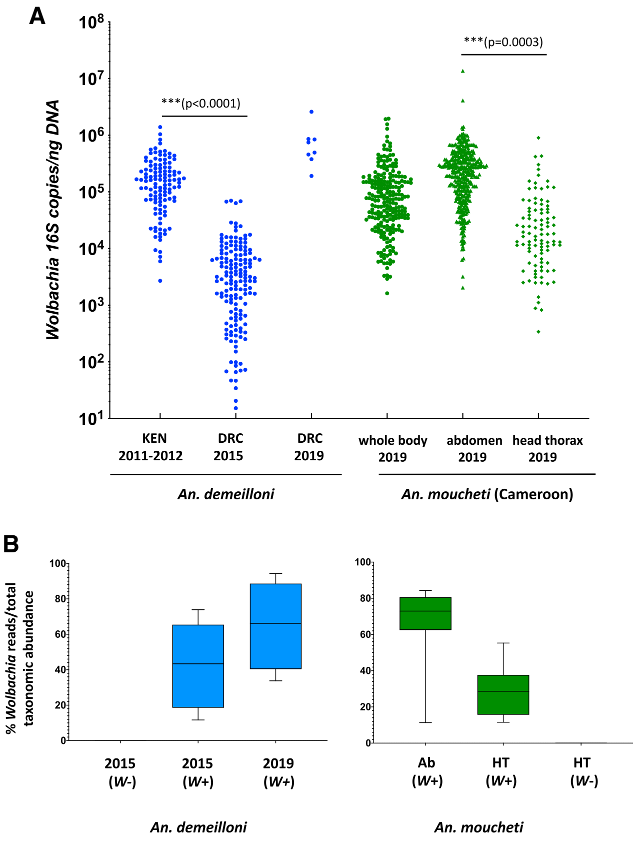

Figure 3. *Wolbachia* strain densities and relative abundance in the mosquito microbiome

(A) Normalized *Wolbachia* strain densities measured using qPCR of the conserved *Wolbachia 16S rRNA* gene. A synthetic oligonucleotide standard was used to calculate *Wolbachia 16S rRNA* gene copies per nanogram of total DNA using a 10-fold serial dilution standard curve. p values from t tests are shown to indicate significant differences.

(B) Relative *Wolbachia* abundance in the mosquito microbiome. Taxonomic abundance of bacterial ASVs within the *16S rRNA* microbiomes of *An. demeilloni* and *An. moucheti* using QIIME 2[24] was used to determine *Wolbachia* percent abundance of total *16S rRNA* bacterial load, indicated through box-and-whisker plots (GraphPad Prism 9).

See also Figure S3.

14 other *Wolbachia* strains sequenced with their hosts (Tables S6, S7, and S8). For *An. demeilloni* and *An. moucheti* samples, the average sequencing depth against *Wolbachia* genomes was comparable to that in mosquitoes such as *Culex* (*Cx.*) *quinquefasciatus* and *Ae. albopictus*, which are known to contain resident *Wolbachia* strains in stable symbiotic associations (Table S4). In contrast, *An. coluzzii* and *An. gambiae* s.s. (including from Burkina Faso)[11] showed exceedingly low sequencing depth against *Wolbachia* genomes, despite high sequencing depth against mosquito genomes.

*w*AnD and *w*AnM genome characteristics and the presence of CI genes

These two newly sequenced genomes share key properties with other *Wolbachia* genomes, including genome size, predicted number of coding sequences, and GC content (Figure 5B; Table S5). Comparative average nucleotide identity (ANI) analysis was undertaken with 48 published *Wolbachia* genomes to reveal that *w*AnD and *w*AnM are closely related to one another, in comparison to the other available genomes (Figure 6; Tables S6, S7, and S8). We also included an assembled *Wolbachia* genome that resulted from a recent large-scale computational study[25] utilizing sequencing data generated in the course of the Ag1000G project, a large international effort determining the *An. gambiae* genome population dynamics.[26] The host species was subsequently classified as *An. species* A[20] (here, we have identified this species as *An. demeilloni*), and this genome shows close to 100% similarity to our assembled *w*AnD genome based on ANI analysis. The *w*AnD and *w*AnM strains cluster with other *Wolbachia* Supergroup B strains (Figure 6), confirming the phylogenetic position indicated by MLST. We analyzed the genomes for evidence of *cif* genes associated with the CI phenotype in other *Wolbachia* strains.[27–29] The *cifA* and *cifB* genes (and corresponding homologs) are neighboring genes found across all CI-inducing strains and group into four monophyletic types.[28,30] We identified two sets of *cif* gene homologs within the genome of *w*AnD, one of which, however, encodes multiple stop-codon and frameshift interruptions (Figure 7). The predicted protein domains, as observed in previous studies,[30] included two PDDEXK nuclease domains, which are a consistent feature across all identified *cifB* genes. In contrast to *w*AnD, the *w*AnM genome contained only one pair of *cif* genes, with the *cifB* gene interrupted with one stop codon and frameshift (Figure 7).

DISCUSSION

Before this study, significant evidence of a stable association between *Anopheles* mosquitoes and endosymbiotic *Wolbachia* bacteria has been lacking.[20] Criticism of previous studies is mainly based on their limitation to highly sensitive nested PCR to amplify *Wolbachia* DNA, which was extrapolated to indicate an endosymbiotic association.[20,21] To date, approaches that show the presence of live bacteria (such as microscopy) rather than the detection of DNA sequences have not been undertaken. Previously, the low infection frequencies and high variation in the *Wolbachia* gene sequences of strains detected from *Anopheles* could be argued to be more consistent with environmental contamination rather than a stable bacterial endosymbiont that undergoes vertical transmission. Furthermore, the presence of highly variable gene sequences within the same mosquito species (particularly in the conserved *16S rRNA* gene) is inconsistent with well-characterized *Wolbachia*-host endosymbiotic associations. Our data presented here provides compelling evidence demonstrating that *An. moucheti* and *An. demeilloni* harbor high-density maternally transmitted *Wolbachia* strains. Our analysis also highlights that there is currently no strong evidence for stable native *Wolbachia* strains in the *An. gambiae* complex.

It could be expected that stable *Wolbachia* strain infections would be found in both geographically and temporally distinct populations of the same mosquito species. Our phylogeographic sequencing data (MLST and *wsp* gene profiles) show that both *w*AnM and *w*AnD strains, derived from their same respective host species, span across large geographical areas and distinct sampling time points, which would be consistent with stably inherited CI-inducing strains. Prevalence rates in wild mosquito

CellPress
OPEN ACCESS

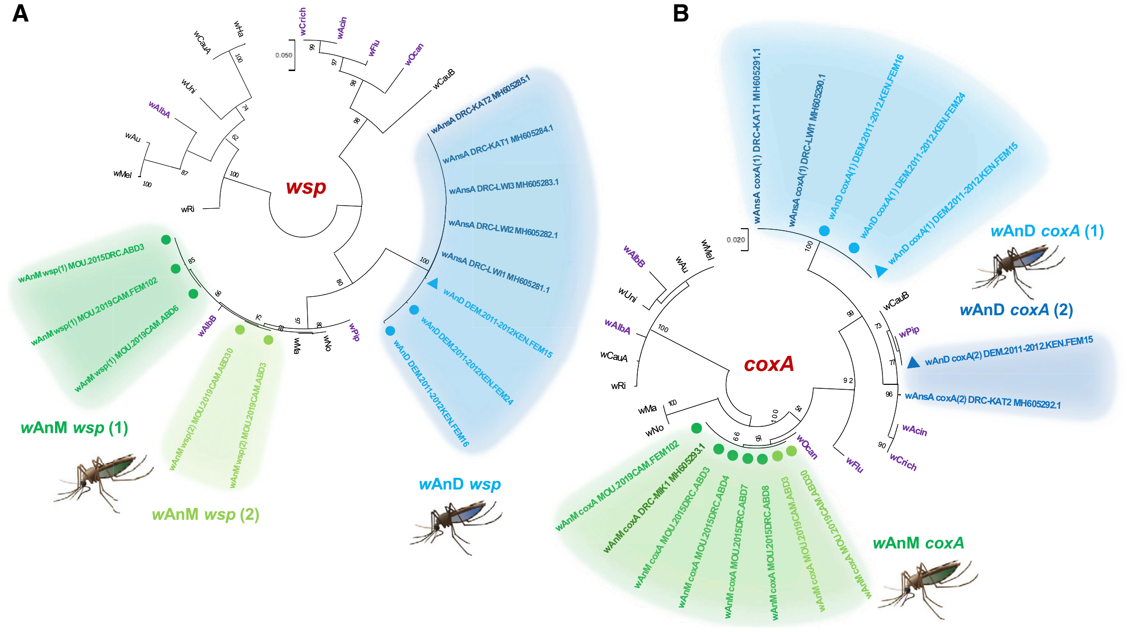

Figure 4. Molecular phylogenetic analyses of *Wolbachia* strains

(A) *Wolbachia* surface protein (*wsp*) phylogenetic tree. The tree with the highest log likelihood (−3,048.85) is indicated, and the analysis involved 29 nucleotide sequences. There were 583 positions in the final dataset. Identical *w*AnM *wsp* sequences from *An. m. moucheti* in Cameroon and the DRC are indicated in dark green. *Wolbachia w*AnM *wsp* variants between *An. m. moucheti* (wsp-1) (dark green) and *An. m. cf. moucheti* (wsp-2) (light green) are also indicated. Identical *w*AnsA/*w*AnD *wsp* sequences obtained from Kenya and the DRC are indicated in blue.

(B) *Wolbachia* cytochrome *c* oxidase subunit I (*coxA*) phylogenetic tree. The tree with the highest log likelihood (−1,208.78) is indicated, with the analysis involving 30 nucleotide sequences and 402 positions. *Wolbachia w*AnsA/*w*AnD *coxA* variants from both Kenya and the DRC are indicated, with identical *coxA*-1 sequences (light blue grouping) and closely grouping *coxA*-2 variants (dark blue) from both countries. The sequences obtained from an *An. demeilloni* specimen in which both strain variants (*w*AnD *coxA*-1 and *coxA*-2) were present are denoted with triangle node markers. *Wolbachia* strains from other mosquito hosts are indicated in purple in both trees.

See also Table S3.

populations are also consistent with CI-inducing strains, and this is in direct contrast to the majority of studies that find a low prevalence rate of detected *Wolbachia* DNA in the *An. gambiae* complex. Further studies are needed to determine whether genetic diversity within the *An. moucheti* complex could be influencing *Wolbachia* prevalence rates and how *Wolbachia* strain variation relates to genetic divergence within the *An. moucheti* complex, as indicated by our *COII* and corresponding *wsp* phylogenetic analysis. Interestingly, sequencing of the *w*AnM genome revealed an interrupted *cifB* gene that could also be indicative of variation in the levels of CI being induced by this strain. Alternatively, intermediate prevalence rates could result from imperfect maternal transmission or fitness costs associated with infection. Further experiments are needed to determine the parameters that influence the ability of these *Wolbachia* strains to invade or be lost from mosquito populations.

To demonstrate the presence of live bacteria, we also provide microscopic data showing intact *Wolbachia* cells in *Anopheles* ovaries using FISH. We show heavily infected ovarian follicles that are comparable to stable infection in the germline of naturally or artificially infected *Aedes*.[31,32] The punctate infection can be seen within the nurse cells that surround the oocyte, which is often seen in *Wolbachia* infections in Diptera. These high-density ovarian infections are in contrast to the low levels of *Wolbachia* observed in *An. coluzzii* and our previous attempts to artificially infect *An. gambiae* s.s., where small punctate infections were seen proximal to the follicular epithelium.[13,33,34]

The densities of *Wolbachia* strains in the *An. gambiae* complex and *An. funestus* are mostly reported at threshold detection levels requiring nested PCR and providing only incomplete MLST profiles.[12,14,15] A recent study using *16S rRNA* gene sequencing of nested-PCR-positive *An. coluzzii* from Burkina Faso found only one mosquito with 42 *Wolbachia* reads constituting 0.04% relative abundance of the microbiome.[35] The inability to find *Wolbachia* reads targeting the *16S rRNA* hypervariable V3–V4 region in nested-PCR-positive individuals raises concerns about the validity of nested PCR, which has been commonly used to report the detection of *Wolbachia* infections in *Anopheles*.[12–17] In comparison, our microbiome analysis shows that, when present, both the *w*AnM and *w*AnD strains dominate the microbiome, which would be more consistent with a maternally transmitted endosymbiont. Furthermore, the inability to amplify and sequence the *wsp* gene from strains detected in the *An. gambiae* complex is also inconsistent with well-characterized *Wolbachia* strains with stable host associations, given that it is a commonly used marker for strain typing (despite having a high rate of recombination) and is approximately 10 times more variable than the *16S rRNA* gene.[36] In contrast, our qPCR and strain typing results (including *wsp*) on larger cohorts

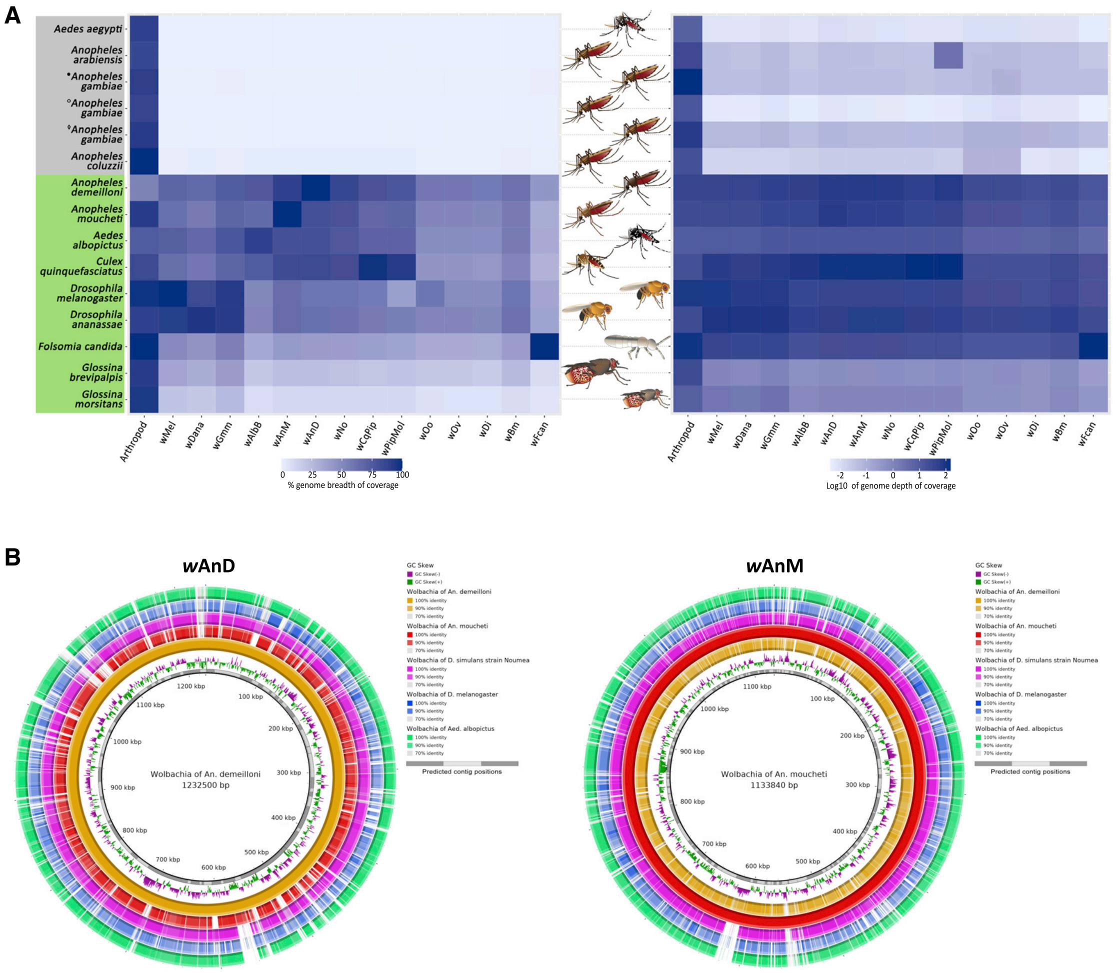

Figure 5. Breadth and depth of coverage of *Wolbachia* genomes

(A) Heatmap of coverage from published genome sequencing datasets after first mapping to the associated host genome and, subsequently, to a selection of *Wolbachia* genomes. Insect hosts without a known native stable *Wolbachia* strain infection are highlighted in gray, while those with a known *Wolbachia* infection are highlighted in green. Analysis includes *An. gambiae* s.l. from previously published studies: (●) Burkina Faso from Baldini et al.,[11] (○) newly sequenced *An. gambiae* from the DRC, and (◇) *An. coluzzii* from Ghana samples sequenced during our study. Shades of dark blue represent high values of either depth or breadth of coverage. Samples from arthropods not known to contain *Wolbachia* have comparatively low depth and breadth of coverage against *Wolbachia* genomes.

(B) Similarities and depth of coverage of *Wolbachia* *w*AnD and *w*AnM genomes compared against a selection of other *Wolbachia* genomes. The BLAST Ring Image Generator (BRIG) program was used to analyze the percentage identity of the *w*AnD and *w*AnM genomes against 5 other *Wolbachia* genomes, including the genomes themselves. Each colored ring from the center represents a different *Wolbachia* genome as represented in the key at the top right of the image, with the saturation of color at certain coordinates of the circle representing how conserved that region of the *w*AnD or *w*AnM genome is when compared against the target *Wolbachia* genome.

See also Tables S4, S5, S6, and S7.

of *An. moucheti* and *An. demeilloni* reinforce that the *w*AnM and *w*AnD strains are present at significantly higher densities.

Finally, evidence for high-density *Wolbachia* infections is further confirmed by the assembly of near-complete genomes. In addition to this, read depths against the assembled genomes were comparable to those of other arthropods with known *Wolbachia* infections. A high genome depth and coverage for both *w*AnM and *w*AnD *Wolbachia* genomes was seen even after sequencing through the more abundant host reads. This is in stark contrast to *An. gambiae* complex sequencing data, in which the very low coverage is comparable to that of insects not known to harbor native *Wolbachia* strains, and mapped reads are likely to represent background noise.[11,20]

Our reported high-density strains that localize in the germline appear desirable for vector control. The two genes responsible for *Wolbachia*-induced sperm modification and rescue (*cifA* and *cifB*) resulting in the CI phenotype were previously identified as part of prophage regions,[28,37,38] and our genome analysis provides strong evidence for the presence of *cif* gene homologs.[39] CI induction would be consistent with both high prevalence rates in wild mosquito populations and maternal transmission and would be desirable for transinfection into other

CellPress
OPEN ACCESS

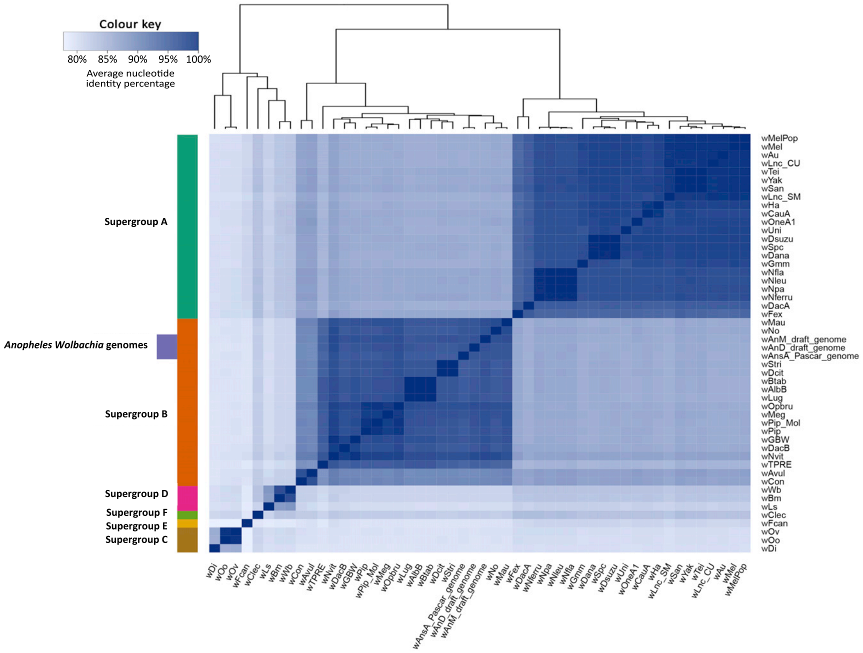

Figure 6. FastANI values and genome clustering analysis

Heatmap indicating the results of FastANI, comparing a total of 48 *Wolbachia* genomes against each other for similarity. High values represent close genetic similarity and a smaller phylogenetic distance, and vice versa with low values, as indicated by the color key at the top left of the heatmap. The color bar at the left of the heatmap indicates previously known clade organization of the analyzed *Wolbachia* species.
See also Tables S8 and S9.

medically relevant *Anopheles* species. Although we did not observe *Wolbachia* in other tissues with microscopy, our qPCR data indicate somatic infection in some individuals. Whether the presence of these two high-density *Wolbachia* strains would affect *Plasmodium* infection remains to be determined, but lower density strains in the *An. gambiae* complex (if genuine endosymbionts) are correlated with *Plasmodium* inhibition.[13,14] Although *Wolbachia* density is important for inhibition of viral pathogens in *Aedes* mosquitoes, the role of density is less clear for *Wolbachia-Plasmodium* interactions.[40,41]

Further characterization of the *w*AnM and *w*AnD strains and their ability to inhibit *Plasmodium* will provide the basis for use in strategies to impact malaria transmission in wild mosquito populations. If further investigation finds that these strains are not ubiquitous across populations of their native host species, then these strains could potentially be utilized in control strategies through the release of *Wolbachia*-infected males for population suppression.[1] Alternatively, if these strains are shown to inhibit *Plasmodium* transmission in their native hosts (highly likely, given that strain inhibition was reported in the *An. gambiae* complex),[13,14] then colony generation followed by selective releases in areas with a lower *Wolbachia* prevalence in wild populations could be undertaken in population replacement strategies. Our work has demonstrated that there is very little evidence for genuine *Wolbachia* strains present in the *An. gambiae* complex, opening up the possibility for transinfection of these high-density strains into these major vector species that are responsible for malaria transmission in much of Sub-Saharan Africa. *Wolbachia* strains from closer phylogenetic host species may be advantageous, as intracellular adaptation to the target host genetic background likely facilitated successful transinfection in *Ae. aegypti*.[31,42,43] Furthermore, transinfection of resident strains in *Anopheles* may also result in less severe fitness costs than those seen for the *w*AlbB strain in *An. stephensi*.[8,44] Sustainable malaria biocontrol using *Wolbachia* bacteria will ultimately require transinfection of strains that both inhibit *Plasmodium* parasites and induce CI without significant fitness costs, and the *w*AnD and *w*AnM strains should be further investigated as candidate strains.

STAR★METHODS

Detailed methods are provided in the online version of this paper and include the following:

- KEY RESOURCES TABLE
- RESOURCE AVAILABILITY
  - Lead contact
  - Materials availability
  - Data and code availability
- EXPERIMENTAL MODEL AND SUBJECT DETAILS
- METHOD DETAILS
  - Study sites, collection methods and historical sample collections
  - DNA extraction and molecular mosquito species identification
  - Wolbachia detection, quantification and confirmation of strain types
  - Multilocus strain typing (MLST)
  - Phylogenetic analysis
  - Microbiome analysis
  - Fluorescent *in situ* hybridization (FISH)
  - Genome sequencing
  - Genome annotation and comparisons to existing genomes and sequence data
  - Genome mapping comparison
- QUANTIFICATION AND STATISTICAL ANALYSIS

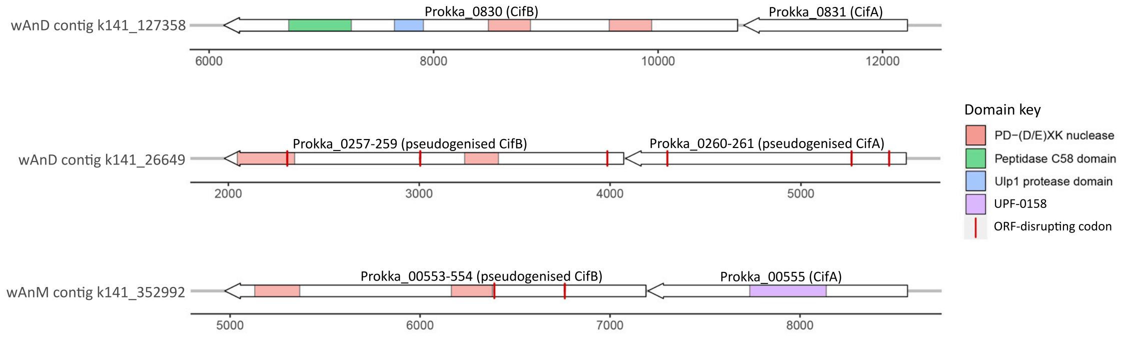

Figure 7. Representation of *cif* genes in the *Wolbachia* genomes

The *cif* genes within the assembled *Wolbachia* genomes are indicated with predicted protein domains overlaid. Each gene pair is drawn in relation to the contig they have been annotated on (x axis, nucleotides). Domains were detected using the HHPred webserver.

SUPPLEMENTAL INFORMATION

Supplemental information can be found online at https://doi.org/10.1016/j.cub.2021.03.056.

ACKNOWLEDGMENTS

The authors would like to thank Ralph Harbach of the Natural History Museum (London, UK) for independent expert morphological identification of mosquito samples and the following people from the Centers for Disease Control and Prevention (CDC) who reviewed our manuscript: Barb Marston, Peter McElroy, B.K. Kapella, and John Gimnig. The authors would also like to thank the staff from CRSN-Lwiro for their help with larval collections. T.W. and C.L.J. were supported by a Sir Henry Dale Wellcome Trust/Royal Society fellowship awarded to T.W. (101285): https://wellcome.org and https://royalsociety.org. G.L.H. was supported by the BBSRC (BB/T001240/1), a Royal Society Wolfson fellowship (RSWF\R1\180013), the NIH (grants R21AI124452 and R21AI129507), the UKRI (20197), and the National Institute for Health Research (NIHR) (NIHR2000907). G.L.H. is affiliated with the NIHR Health Protection Research Unit (NIHR HPRU) in Emerging and Zoonotic Infections at the University of Liverpool in partnership with Public Health England (PHE), in collaboration with the Liverpool School of Tropical Medicine (LSTM) and the University of Oxford. G.L.H. is based at LSTM. G.L.H. and E.H. are also jointly funded by the BBSRC (V011278/1). S.H. was supported by the LSTM Director's Catalyst Fund award. The views expressed are those of the author(s) and not necessarily those of the NHS, the NIHR, the Department of Health, Public Health England, or the Centers for Disease Control (CDC) and Prevention.

AUTHOR CONTRIBUTIONS

T.W. acquired funding, supervised mosquito field collections, undertook laboratory analysis of samples, provided overall supervision alongside G.L.H., and co-wrote the first draft. S.Q. undertook genome assemblies and genome analysis and co-wrote the first draft. C.L.J. undertook laboratory analysis of samples, Sanger sequencing and phylogenetic analysis, microbiome analysis, and supervision of field collections and co-wrote the first draft. J.B. undertook mosquito colonization and provided samples from the DRC. V.D. contributed to mosquito collection, colonization and laboratory analysis of samples. R.B. contributed to mosquito collection and colonization. M.K. contributed to laboratory analysis of samples and microbiome analysis. L.A.M. contributed to laboratory analysis of samples and microbiome analysis. A.G. contributed to mosquito collection and laboratory analysis of samples. E.A.H. contributed to microbiome analysis. E.R.A. undertook FISH. C.C.-U. contributed to figure preparation. S.H. contributed to FISH. C.B. provided reagents and support for mosquito colonization in the DRC. J.C.S. and N.F.L. contributed mosquito DNA samples from Kenya. S.C.W. contributed to *Wolbachia* genomic sequencing analysis. C.A.N. provided logistical support and supervision of fieldwork in Cameroon. S.R.I. provided mosquito and DNA samples from the DRC and undertook morphological identification. E.H. supervised *Wolbachia* genome sequence analysis and co-wrote the first draft. G.L.H. acquired funding, supervised FISH, and provided overall supervision alongside T.W. and co-wrote the first draft.

DECLARATION OF INTERESTS

The authors declare no competing interests.

Received: December 17, 2020
Revised: February 15, 2021
Accepted: March 16, 2021
Published: April 14, 2021

REFERENCES

1. Zheng, X., Zhang, D., Li, Y., Yang, C., Wu, Y., Liang, X., Liang, Y., Pan, X., Hu, L., Sun, Q., et al. (2019). Incompatible and sterile insect techniques combined eliminate mosquitoes. Nature *572*, 56–61.
2. Crawford, J.E., Clarke, D.W., Criswell, V., Desnoyer, M., Cornel, D., Deegan, B., Gong, K., Hopkins, K.C., Howell, P., Hyde, J.S., et al. (2020). Efficient production of male Wolbachia-infected Aedes aegypti mosquitoes enables large-scale suppression of wild populations. Nat. Biotechnol. *38*, 482–492.
3. Indriani, C., Tantowijoyo, W., Rancès, E., Andari, B., Prabowo, E., Yusdi, D., Ansari, M.R., Wardana, D.S., Supriyati, E., Nurhayati, I., et al. (2020). Reduced dengue incidence following deployments of *Wolbachia*-infected *Aedes aegypti* in Yogyakarta, Indonesia: a quasi-experimental trial using controlled interrupted time series analysis. Gates Open Res. *4*, 50.
4. Hoffmann, A.A., Montgomery, B.L., Popovici, J., Iturbe-Ormaetxe, I., Johnson, P.H., Muzzi, F., Greenfield, M., Durkan, M., Leong, Y.S., Dong, Y., et al. (2011). Successful establishment of Wolbachia in Aedes populations to suppress dengue transmission. Nature *476*, 454–457.
5. Nazni, W.A., Hoffmann, A.A., NoorAfizah, A., Cheong, Y.L., Mancini, M.V., Golding, N., Kamarul, G.M.R., Arif, M.A.K., Thohir, H., NurSyamimi, H., et al. (2019). Establishment of Wolbachia strain wAlbB in Malaysian populations of Aedes aegypti for dengue control. Curr. Biol. *29*, 4241–4248.e5.
6. Moyes, C.L., Athinya, D.K., Seethaler, T., Battle, K.E., Sinka, M., Hadi, M.P., Hemingway, J., Coleman, M., and Hancock, P.A. (2020). Evaluating insecticide resistance across African districts to aid malaria control decisions. Proc. Natl. Acad. Sci. USA *117*, 22042–22050.
7. Hughes, G.L., Koga, R., Xue, P., Fukatsu, T., and Rasgon, J.L. (2011). Wolbachia infections are virulent and inhibit the human malaria parasite Plasmodium falciparum in Anopheles gambiae. PLoS Pathog. *7*, e1002043.

CellPress
OPEN ACCESS

8. Bian, G., Joshi, D., Dong, Y., Lu, P., Zhou, G., Pan, X., Xu, Y., Dimopoulos, G., and Xi, Z. (2013). Wolbachia invades Anopheles stephensi populations and induces refractoriness to Plasmodium infection. Science *340*, 748–751.
9. Hughes, G.L., and Rasgon, J.L. (2014). Transinfection: a method to investigate Wolbachia-host interactions and control arthropod-borne disease. Insect Mol. Biol. *23*, 141–151.
10. Walker, T., and Moreira, L.A. (2011). Can Wolbachia be used to control malaria? Mem. Inst. Oswaldo Cruz *106* (*Suppl 1*), 212–217.
11. Baldini, F., Segata, N., Pompon, J., Marcenac, P., Shaw, W.R., Dabiré, R.K., Diabaté, A., Levashina, E.A., and Catteruccia, F. (2014). Evidence of natural Wolbachia infections in field populations of Anopheles gambiae. Nat. Commun. *5*, 3985.
12. Baldini, F., Rougé, J., Kreppel, K., Mkandawile, G., Mapua, S.A., Sikulu-Lord, M., Ferguson, H.M., Govella, N., and Okumu, F.O. (2018). First report of natural Wolbachia infection in the malaria mosquito Anopheles arabiensis in Tanzania. Parasit. Vectors *11*, 635.
13. Shaw, W.R., Marcenac, P., Childs, L.M., Buckee, C.O., Baldini, F., Sawadogo, S.P., Dabiré, R.K., Diabaté, A., and Catteruccia, F. (2016). Wolbachia infections in natural Anopheles populations affect egg laying and negatively correlate with Plasmodium development. Nat. Commun. *7*, 11772.
14. Gomes, F.M., Hixson, B.L., Tyner, M.D.W., Ramirez, J.L., Canepa, G.E., Alves E Silva, T.L., Molina-Cruz, A., Keita, M., Kane, F., Traoré, B., et al. (2017). Effect of naturally occurring *Wolbachia* in *Anopheles gambiae s.l.* mosquitoes from Mali on *Plasmodium falciparum* malaria transmission. Proc. Natl. Acad. Sci. USA *114*, 12566–12571.
15. Niang, E.H.A., Bassene, H., Makoundou, P., Fenollar, F., Weill, M., and Mediannikov, O. (2018). First report of natural Wolbachia infection in wild Anopheles funestus population in Senegal. Malar. J. *17*, 408.
16. Wong, M.L., Liew, J.W.K., Wong, W.K., Pramasivan, S., Mohamed Hassan, N., Wan Sulaiman, W.Y., Jeyaprakasam, N.K., Leong, C.S., Low, V.L., and Vythilingam, I. (2020). Natural Wolbachia infection in field-collected Anopheles and other mosquito species from Malaysia. Parasit. Vectors *13*, 414.
17. Ayala, D., Akone-Ella, O., Rahola, N., Kengne, P., Ngangue, M.F., Mezeme, F., Makanga, B.K., Nigg, M., Costantini, C., Simard, F., et al. (2019). Natural *Wolbachia* infections are common in the major malaria vectors in Central Africa. Evol. Appl. *12*, 1583–1594.
18. Jeffries, C.L., Lawrence, G.G., Golovko, G., Kristan, M., Orsborne, J., Spence, K., Hurn, E., Bandibabone, J., Tantely, L.M., Raharimalala, F.N., et al. (2018). Novel *Wolbachia* strains in *Anopheles* malaria vectors from Sub-Saharan Africa. Wellcome Open Res. *3*, 113.
19. Carini, P., Marsden, P.J., Leff, J.W., Morgan, E.E., Strickland, M.S., and Fierer, N. (2016). Relic DNA is abundant in soil and obscures estimates of soil microbial diversity. Nat. Microbiol. *2*, 16242.
20. Chrostek, E., and Gerth, M. (2019). Is Anopheles gambiae a natural host of *Wolbachia?* MBio *10*, e00784-19.
21. Ross, P.A., Callahan, A.G., Yang, Q., Jasper, M., Arif, M.A.K., Afizah, A.N., Nazni, W.A., and Hoffmann, A.A. (2020). An elusive endosymbiont: does *Wolbachia* occur naturally in *Aedes aegypti?* Ecol. Evol. *10*, 1581–1591.
22. Jeffries, C.L., Cansado-Utrilla, C., Beavogui, A.H., Stica, C., Lama, E.K., Kristan, M., et al. (2021). Evidence for natural hybridization and novel *Wolbachia* strain superinfections in the *Anopheles gambiae* complex from Guinea. R. Soc. Open Sci. *8*, 202032.
23. Antonio-Nkondjio, C., Ndo, C., Kengne, P., Mukwaya, L., Awono-Ambene, P., Fontenille, D., and Simard, F. (2008). Population structure of the malaria vector Anopheles moucheti in the equatorial forest region of Africa. Malar. J. *7*, 120.
24. Bolyen, E., Rideout, J.R., Dillon, M.R., Bokulich, N.A., Abnet, C.C., Al-Ghalith, G.A., Alexander, H., Alm, E.J., Arumugam, M., Asnicar, F., et al. (2019). Reproducible, interactive, scalable and extensible microbiome data science using QIIME 2. Nat. Biotechnol. *37*, 852–857.
25. Pascar, J., and Chandler, C.H. (2018). A bioinformatics approach to identifying *Wolbachia* infections in arthropods. PeerJ *6*, e5486.
26. The Anopheles gambiae 1000 Genomes Consortium (2017). Genetic diversity of the African malaria vector Anopheles gambiae. Nature *552*, 96–100.
27. Beckmann, J.F., Ronau, J.A., and Hochstrasser, M. (2017). A Wolbachia deubiquitylating enzyme induces cytoplasmic incompatibility. Nat. Microbiol. *2*, 17007.
28. LePage, D.P., Metcalf, J.A., Bordenstein, S.R., On, J., Perlmutter, J.I., Shropshire, J.D., Layton, E.M., Funkhouser-Jones, L.J., Beckmann, J.F., and Bordenstein, S.R. (2017). Prophage WO genes recapitulate and enhance Wolbachia-induced cytoplasmic incompatibility. Nature *543*, 243–247.
29. Bonneau, M., Atyame, C., Beji, M., Justy, F., Cohen-Gonsaud, M., Sicard, M., and Weill, M. (2018). Culex pipiens crossing type diversity is governed by an amplified and polymorphic operon of Wolbachia. Nat. Commun. *9*, 319.
30. Lindsey, A.R.I., Rice, D.W., Bordenstein, S.R., Brooks, A.W., Bordenstein, S.R., and Newton, I.L.G. (2018). Evolutionary genetics of cytoplasmic incompatibility genes cifA and cifB in prophage WO of Wolbachia. Genome Biol. Evol. *10*, 434–451.
31. Walker, T., Johnson, P.H., Moreira, L.A., Iturbe-Ormaetxe, I., Frentiu, F.D., McMeniman, C.J., Leong, Y.S., Dong, Y., Axford, J., Kriesner, P., et al. (2011). The wMel Wolbachia strain blocks dengue and invades caged Aedes aegypti populations. Nature *476*, 450–453.
32. Joubert, D.A., Walker, T., Carrington, L.B., De Bruyne, J.T., Kien, D.H., Hoang, Nle.T., Chau, N.V., Iturbe-Ormaetxe, I., Simmons, C.P., and O'Neill, S.L. (2016). Establishment of a Wolbachia superinfection in Aedes aegypti mosquitoes as a potential approach for future resistance management. PLoS Pathog. *12*, e1005434.
33. Hughes, G.L., Dodson, B.L., Johnson, R.M., Murdock, C.C., Tsujimoto, H., Suzuki, Y., Patt, A.A., Cui, L., Nossa, C.W., Barry, R.M., et al. (2014). Native microbiome impedes vertical transmission of Wolbachia in Anopheles mosquitoes. Proc. Natl. Acad. Sci. USA *111*, 12498–12503.
34. Hughes, G.L., Pike, A.D., Xue, P., and Rasgon, J.L. (2012). Invasion of Wolbachia into Anopheles and other insect germlines in an ex vivo organ culture system. PLoS ONE *7*, e36277.
35. Straub, T.J., Shaw, W.R., Marcenac, P., Sawadogo, S.P., Dabiré, R.K., Diabaté, A., Catteruccia, F., and Neafsey, D.E. (2020). The Anopheles coluzzii microbiome and its interaction with the intracellular parasite Wolbachia. Sci. Rep. *10*, 13847.
36. Zhou, W., Rousset, F., and O'Neil, S. (1998). Phylogeny and PCR-based classification of Wolbachia strains using wsp gene sequences. Proc. Biol. Sci. *265*, 509–515.
37. Bordenstein, S.R., and Bordenstein, S.R. (2016). Eukaryotic association module in phage WO genomes from Wolbachia. Nat. Commun. *7*, 13155.
38. Shropshire, J.D., On, J., Layton, E.M., Zhou, H., and Bordenstein, S.R. (2018). One prophage WO gene rescues cytoplasmic incompatibility in *Drosophila melanogaster*. Proc. Natl. Acad. Sci. USA *115*, 4987–4991.
39. Martinez, J., Klasson, L., Welch, J.J., and Jiggins, F.M. (2020). Life and death of selfish genes: comparative genomics reveals the dynamic evolution of cytoplasmic incompatibility. Mol. Biol. Evol. *38*, 2–15.
40. Zélé, F., Nicot, A., Berthomieu, A., Weill, M., Duron, O., and Rivero, A. (2014). Wolbachia increases susceptibility to Plasmodium infection in a natural system. Proc Biol Sci. *281* (1779), 20132837.
41. Hughes, G.L., Vega-Rodriguez, J., Xue, P., and Rasgon, J.L. (2012). Wolbachia strain wAlbB enhances infection by the rodent malaria parasite Plasmodium berghei in Anopheles gambiae mosquitoes. Appl. Environ. Microbiol. *78*, 1491–1495.
42. McMeniman, C.J., Lane, R.V., Cass, B.N., Fong, A.W., Sidhu, M., Wang, Y.F., and O'Neill, S.L. (2009). Stable introduction of a life-shortening Wolbachia infection into the mosquito Aedes aegypti. Science *323*, 141–144.
43. McMeniman, C.J., Lane, A.M., Fong, A.W., Voronin, D.A., Iturbe-Ormaetxe, I., Yamada, R., McGraw, E.A., and O'Neill, S.L. (2008). Host adaptation of a Wolbachia strain after long-term serial passage in mosquito cell lines. Appl. Environ. Microbiol. *74*, 6963–6969.

44. Joshi, D., McFadden, M.J., Bevins, D., Zhang, F., and Xi, Z. (2014). Wolbachia strain wAlbB confers both fitness costs and benefit on Anopheles stephensi. Parasit. Vectors *7*, 336.

45. Heddi, A., Grenier, A.M., Khatchadourian, C., Charles, H., and Nardon, P. (1999). Four intracellular genomes direct weevil biology: nuclear, mitochondrial, principal endosymbiont, and Wolbachia. Proc. Natl. Acad. Sci. USA *96*, 6814–6819.

46. Kumar, S., Stecher, G., Li, M., Knyaz, C., and Tamura, K. (2018). MEGA X: molecular evolutionary genetics analysis across computing platforms. Mol. Biol. Evol. *35*, 1547–1549.

47. Baldo, L., Dunning Hotopp, J.C., Jolley, K.A., Bordenstein, S.R., Biber, S.A., Choudhury, R.R., Hayashi, C., Maiden, M.C., Tettelin, H., and Werren, J.H. (2006). Multilocus sequence typing system for the endosymbiont Wolbachia pipientis. Appl. Environ. Microbiol. *72*, 7098–7110.

48. Martin, M. (2011). Cutadapt removes adapter sequences from high-throughput sequencing reads. EMBnet.journal *17*, 10–12.

49. Callahan, B.J., McMurdie, P.J., Rosen, M.J., Han, A.W., Johnson, A.J., and Holmes, S.P. (2016). DADA2: high-resolution sample inference from Illumina amplicon data. Nat. Methods *13*, 581–583.

50. Bokulich, N.A., Kaehler, B.D., Rideout, J.R., Dillon, M., Bolyen, E., Knight, R., Huttley, G.A., and Caporaso, J.G. (2018). Optimizing taxonomic classification of marker-gene amplicon sequences with QIIME 2's q2-feature-classifier plugin. Microbiome *6*, 90.

51. Quast, C., Pruesse, E., Yilmaz, P., Gerken, J., Schweer, T., Yarza, P., Peplies, J., and Glöckner, F.O. (2013). The SILVA ribosomal RNA gene database project: improved data processing and web-based tools. Nucleic Acids Res. *41*, D590–D596.

52. Bolger, A.M., Lohse, M., and Usadel, B. (2014). Trimmomatic: a flexible trimmer for Illumina sequence data. Bioinformatics *30*, 2114–2120.

53. Giraldo-Calderón, G.I., Emrich, S.J., MacCallum, R.M., Maslen, G., Dialynas, E., Topalis, P., Ho, N., Gesing, S., Madey, G., Collins, F.H., and Lawson, D.; VectorBase Consortium (2015). VectorBase: an updated bioinformatics resource for invertebrate vectors and other organisms related with human diseases. Nucleic Acids Res. *43*, D707–D713.

54. Li, H., and Durbin, R. (2009). Fast and accurate short read alignment with Burrows-Wheeler transform. Bioinformatics *25*, 1754–1760.

55. Li, D., Liu, C.M., Luo, R., Sadakane, K., and Lam, T.W. (2015). MEGAHIT: an ultra-fast single-node solution for large and complex metagenomics assembly via succinct de Bruijn graph. Bioinformatics *31*, 1674–1676.

56. Mikheenko, A., Saveliev, V., and Gurevich, A. (2016). MetaQUAST: evaluation of metagenome assemblies. Bioinformatics *32*, 1088–1090.

57. Darling, A.C., Mau, B., Blattner, F.R., and Perna, N.T. (2004). Mauve: multiple alignment of conserved genomic sequence with rearrangements. Genome Res. *14*, 1394–1403.

58. Rissman, A.I., Mau, B., Biehl, B.S., Darling, A.E., Glasner, J.D., and Perna, N.T. (2009). Reordering contigs of draft genomes using the Mauve aligner. Bioinformatics *25*, 2071–2073.

59. Li, H., Handsaker, B., Wysoker, A., Fennell, T., Ruan, J., Homer, N., Marth, G., Abecasis, G., and Durbin, R.; 1000 Genome Project Data Processing Subgroup (2009). The Sequence Alignment/Map format and SAMtools. Bioinformatics *25*, 2078–2079.

60. Walker, B.J., Abeel, T., Shea, T., Priest, M., Abouelliel, A., Sakthikumar, S., Cuomo, C.A., Zeng, Q., Wortman, J., Young, S.K., and Earl, A.M. (2014). Pilon: an integrated tool for comprehensive microbial variant detection and genome assembly improvement. PLoS ONE *9*, e112963.

61. Seemann, T. (2014). Prokka: rapid prokaryotic genome annotation. Bioinformatics *30*, 2068–2069.

62. Parks, D.H., Imelfort, M., Skennerton, C.T., Hugenholtz, P., and Tyson, G.W. (2015). CheckM: assessing the quality of microbial genomes recovered from isolates, single cells, and metagenomes. Genome Res. *25*, 1043–1055.

63. Jain, C., Rodriguez-R, L.M., Phillippy, A.M., Konstantinidis, K.T., and Aluru, S. (2018). High throughput ANI analysis of 90K prokaryotic genomes reveals clear species boundaries. Nat. Commun. *9*, 5114.

64. Warnes, G.R., Bolker, B.M., Bonebakker, L., Gentleman, R., Huber, W., Liaw, A., Lumley, T., Maechler, M., Magnusson, A., Moeller, S., et al. (2020). Gplots: various R programming tools for plotting data.

65. Zimmermann, L., Stephens, A., Nam, S.Z., Rau, D., Kübler, J., Lozajic, M., Gabler, F., Söding, J., Lupas, A.N., and Alva, V. (2018). A completely reimplemented MPI bioinformatics toolkit with a new HHpred server at its core. J. Mol. Biol. *430*, 2237–2243.

66. Wickham, H. (2016). ggplot2: Elegant Graphics for Data Analysis (Springer-Verlag).

67. Alikhan, N.F., Petty, N.K., Ben Zakour, N.L., and Beatson, S.A. (2011). BLAST Ring Image Generator (BRIG): simple prokaryote genome comparisons. BMC Genomics *12*, 402.

68. Quinlan, A.R., and Hall, I.M. (2010). BEDTools: a flexible suite of utilities for comparing genomic features. Bioinformatics *26*, 841–842.

69. Bamou, R., Mbakop, L.R., Kopya, E., Ndo, C., Awono-Ambene, P., Tchuinkam, T., Rono, M.K., Mwangangi, J., and Antonio-Nkondjio, C. (2018). Changes in malaria vector bionomics and transmission patterns in the equatorial forest region of Cameroon between 2000 and 2017. Parasit. Vectors *11*, 464.

70. Gillies, M., and De Meillon, B. (1968). The Anophelinae of Africa South of the Sahara (South African Institute for Medical Research).

71. Bandibabone, J., Muhigwa, J.B., Agramonte, N.M., Zawadi, B., Ombeni, L., Corredor-Medina, C., Lawrence, G.G., Chimanuka, B., and Irish, S.R. (2018). Identification of Anopheles species in Sud Kivu, Democratic Republic of Congo, using molecular tools. Trans. R. Soc. Trop. Med. Hyg. *112*, 405–407.

72. St Laurent, B., Cooke, M., Krishnankutty, S.M., Asih, P., Mueller, J.D., Kahindi, S., Ayoma, E., Oriango, R.M., Thumloup, J., Drakeley, C., et al. (2016). Molecular characterization reveals diverse and unknown malaria vectors in the Western Kenyan Highlands. Am. J. Trop. Med. Hyg. *94*, 327–335.

73. Beebe, N.W., and Saul, A. (1995). Discrimination of all members of the Anopheles punctulatus complex by polymerase chain reaction–restriction fragment length polymorphism analysis. Am. J. Trop. Med. Hyg. *53*, 478–481.

74. Ndo, C., Antonio-Nkondjio, C., Cohuet, A., Ayala, D., Kengne, P., Morlais, I., Awono-Ambene, P.H., Couret, D., Ngassam, P., Fontenille, D., and Simard, F. (2010). Population genetic structure of the malaria vector Anopheles nili in sub-Saharan Africa. Malar. J. *9*, 161.

75. Tamura, K., and Nei, M. (1993). Estimation of the number of nucleotide substitutions in the control region of mitochondrial DNA in humans and chimpanzees. Mol. Biol. Evol. *10*, 512–526.

76. Klindworth, A., Pruesse, E., Schweer, T., Peplies, J., Quast, C., Horn, M., and Glöckner, F.O. (2013). Evaluation of general 16S ribosomal RNA gene PCR primers for classical and next-generation sequencing-based diversity studies. Nucleic Acids Res. *41*, e1.

77. Pedregosa, F., Varoquaux, G., Gramfort, A., Michel, V., Thirion, B., Grisel, O., Blondel, M., Prettenhofer, P., Weiss, R., Dubourg, V., et al. (2011). Scikit-learn: machine learning in Python. J. Mach. Learn. Res. *12*, 2825–2830.

78. Dodson, B.L., Hughes, G.L., Paul, O., Matacchiero, A.C., Kramer, L.D., and Rasgon, J.L. (2014). Wolbachia enhances West Nile virus (WNV) infection in the mosquito Culex tarsalis. PLoS Negl. Trop. Dis. *8*, e2965.

79. Ellegaard, K.M., Klasson, L., Näslund, K., Bourtzis, K., and Andersson, S.G. (2013). Comparative genomics of Wolbachia and the bacterial species concept. PLoS Genet. *9*, e1003381.

CellPress
OPEN ACCESS

STAR★METHODS

KEY RESOURCES TABLE

REAGENT or RESOURCE	SOURCE	IDENTIFIER
**Biological Samples**		
Mosquitoes analyzed in this study	This study	https://doi.org/10.17605/OSF.IO/AHNB6
**Critical Commercial Assays**		
DNeasy Blood and Tissue Kits	QIAGEN	Cat#69582
QuantiNova SYBR Green PCR Kit	QIAGEN	Cat#208056
FastStart SYBR Green Master mix	Roche Diagnostics	Cat#06924204001
Qubit DNA High Sensitivity Assays	Invitrogen	Cat#Q32854
KAPA HiFi HotStart ReadyMix PCR Kit	Roche Diagnostics	Cat#KK2602
D1000 ScreenTape Assay	Agilent	Cat#G2991AA
**Deposited Data**		
Wolbachia and mosquito gene Sanger sequencing	This study	GenBank: MW250655 - MW250767
Raw *Wolbachia* qPCR data	This study	https://doi.org/10.17605/OSF.IO/AHNB6
Raw genome and microbiome sequencing data	This study	NCBI BioProject PRJNA642000
**Oligonucleotides**		
See Table S10	N/A	N/A
**Probes**		
Wol3_Red (/5ATTO590N/TCCTCTATCCTCTTTCAATC)	Heddi et al.[45]	N/A
Wol4_Red (GAGTTAGCCAGGACTTCTTC/3ATTO590N/)	Heddi et al.[45]	N/A
**Software and Algorithms**		
LightCycler 96 software	Roche Diagnostics	https://lifescience.roche.com/en_gb/brands/realtime-pcr-overview.html#software
MEGAX	Kumar et al.[46]	https://www.megasoftware.net
Wolbachia MLST database	Baldo et al.[47]	https://pubmlst.org/wolbachia
QIIME2 Core (q2cli) 2019.7 distribution	Bolyen et al.[24]	https://qiime2.org
q2-cutadapt plugin	Martin[48]	https://github.com/qiime2/q2-cutadapt
q2-dada2 plugin	Callahan et al.[49]	https://github.com/qiime2/q2-dada2
q2-feature-classifier plugin	Bokulich et al.[50]	https://github.com/qiime2/q2-feature-classifier
16S rRNA SILVA SSU v132 97% reference database	Quast et al.[51]	https://www.arb-silva.de/documentation/release-132/
Trimmomatic	Bolger et al.[52]	http://www.usadellab.org/cms/?page=trimmomatic
VectorBase	Giraldo-Calderón et al.[53]	https://vectorbase.org/vectorbase/
BWA aligner	Li and Durbin[54]	http://bio-bwa.sourceforge.net
MEGAHit	Li et al.[55]	https://github.com/voutcn/megahit
MetaQUAST	Mikheenko et al.[56]	http://quast.sourceforge.net/metaquast
Mauve contig mover	Darling et al.[57]; Rissman et al.[58]	http://darlinglab.org/mauve/
samtools depth	Li et al.[59]	http://www.htslib.org/doc/samtools-depth
Pilon	Walker et al.[60]	https://github.com/broadinstitute/pilon/
PROKKA	Seemann[61]	https://github.com/tseemann/prokka
CheckM	Parks et al.[62]	https://ecogenomics.github.io/CheckM/
FastANI	Jain et al.[63]	https://github.com/ParBLiSS/FastANI
gplot's heatmap.2	Warnes et al.[64]	https://biocorecrg.github.io/CRG_RIntroduction/heatmap-2-function-from-gplots-package
HHPred webserver	Zimmermann et al.[65]	https://toolkit.tuebingen.mpg.de/tools/hhpred

(*Continued on next page*)

Continued		
REAGENT or RESOURCE	SOURCE	IDENTIFIER
ggplot2	Wickham[66]	https://ggplot2.tidyverse.org
Blast Ring Image Generator	Alikhan et al.[67]	http://brig.sourceforge.net
BEDTools' genomeCoverageBed	Quinlan and Hall[68]	https://bedtools.readthedocs.io/en/latest/content/overview.html
BEDTools' makewindows and coverage commands	Quinlan and Hall[68]	https://bedtools.readthedocs.io/en/latest/content/overview.html

RESOURCE AVAILABILITY

Lead contact

Further information and requests for resources should be directed to and will be fulfilled by the Lead Contact, Thomas Walker (Thomas.walker@lshtm.ac.uk).

Materials availability

This study did not generate new unique reagents.

Data and code availability

Raw qPCR data is available at https://osf.io/ahnb6/. Raw sequencing data has been uploaded to NCBI under BioProject PRJNA642000, accession numbers SRR12095496 through to SRR12095498, SRR12729562, and SRR12799871 through to SRR12799876. Sanger sequencing data is available with accession numbers as listed in Table S1.

EXPERIMENTAL MODEL AND SUBJECT DETAILS

Individual mosquito sample details including species, collection year and collection location is available at https://osf.io/ahnb6/. All mosquitoes analyzed were collected or provided as DNA extracts by authors of this study. Ethical approval for undertaking Human landing catches (HLCs) in Cameroon was obtained from the LSHTM ethics committee (reference no. 16684) in addition to local ethical approval (clearance no. 2016/01/685/CE/CNERSH/SP) delivered by the Cameroon National Ethics (CNE) Committee for Research on Human Health). Informed consent was gained from all collectors prior to commencement of sampling and all collectors were provided with malarial chemoprophylaxis.

METHOD DETAILS

Study sites, collection methods and historical sample collections

A variety of sampling methods were used to obtain new mosquito collections in selected study sites, in addition to analysis of historical DNA samples. *Anopheles* adult collections were undertaken in Olama Village (3.4125, 11.28416), Cameroon in June-July 2019 (Table S11) as this location has previously shown a high abundance of *An. moucheti*.[69] HLCs were undertaken between 19:00 and 06:00 for a total of 13 nights. In total, 104 Person/Trap/Nights were conducted, with 52 indoors and 52 outdoors. Trained collectors were stationed at each house, with one individual inside and another outside. Participants exposed their legs and were provided with a flashlight. All mosquitoes that landed on exposed legs were collected in clear tubes and sealed with cotton wool. Tubes were organized into cotton bags labeled by hour, house number and location (indoors/outdoors). To reduce individual attraction bias, participants were rotated between houses for each night of collection, and halfway through each collection night the two collectors at each house swapped places. All collection bags were transported from the field back to the Organization de Coordination pour la lutte contre les Endémies en Afrique Centrale (Yaoundé, Cameroon) for morphological identification using keys.[70] Dead *An. moucheti* females were either stored in 100% absolute ethanol for subsequent PCR-based molecular analysis or in 100% acetone after removal of legs and wings to undergo FISH. Early generation colonization was performed at OCEAC and later at LSHTM.

Larval sampling was undertaken in Lwiro (−2.244097, 28.815232), a village near Katana in the Democratic Republic of the Congo (DRC) in March 2019 to supplement existing mosquito DNA samples resulting from a 2015 collection containing a high abundance of *An. species* A individuals.[71] Larvae were collected and colonization was performed at CRSN/LWIRO and later LSHTM. Morphological identification on adult females was independently carried out at LSHTM and CRSN/LWIRO (DRC) following keys.[3,35] Historical DNA samples of *An. species* A were also analyzed from an area of Western Kenya.[72]

DNA extraction and molecular mosquito species identification

Genomic DNA from whole bodies or dissected body parts (head-thorax and abdomens) were individually extracted using QIAGEN DNeasy Blood and Tissue Kits according to manufacturer's instructions. DNA extracts were eluted in a final volume of 100 μL and stored at −20°C. To confirm species identification, a sub-set of individuals from all locations were subject to Sanger sequencing and

phylogenetic analysis of ITS2[73] and *COII*[74] PCR products to enable greater differentiation of specimens. Sanger sequencing of PCR products was carried out as previously described[18] (sequence GenBank accession numbers are listed in Table S1). To generate a rapid method for confirming mosquito species, ITS2 sequences for both *An. moucheti* and *An. demeilloni* were aligned (Figure S1A) and used to design species-specific qPCR assays (Figure S1B). Forward and reverse primer sequences to amplify a fragment of the *An. moucheti* ITS2 were 5′-GTCGCAGGCTTGAACACA-3′ and 5′-ACTGTACCGCCTTACCATTTC-3′ respectively. Forward and reverse primer sequences to amplify a fragment of *An. demeilloni* ITS2 were 5′-GCTTAAGGCAGGTAAGGCGA-3′ and 5′-CGGTGTTAGAAGGCTCCGTT-3′ respectively. qPCR reactions were prepared using 5 μL of FastStart SYBR Green Master mix (Roche Diagnostics) with a final concentration of 1μM of each primer, 1 μL of PCR grade water and 2 μL template DNA, to a final reaction volume of 10 μL. Prepared reactions were run on a Roche LightCycler 96 System for 15 minutes at 95°C, followed by 40 cycles of 95°C for 5 s, 60°C for 5 s and 72°C for 10 s. Amplification was followed by a dissociation curve (95°C for 10 s, 65°C for 60 s and 97°C for 1 s) to ensure the correct target sequence was being amplified.

Wolbachia detection, quantification and confirmation of strain types

Wolbachia detection and quantification was undertaken through qPCR targeting the conserved *Wolbachia 16S rRNA* gene.[14] BLAST analysis and alignments were first performed on previously generated *Wolbachia 16S rRNA* sequences for the *w*AnM and *w*AnD (previously known as *w*AnsA) strains of *Wolbachia*[18] to confirm there was no sequence variability in primer binding regions, which could influence successful amplification. To estimate *Wolbachia* density across multiple *Anopheles* species, DNA extracts were added to Qubit DNA High Sensitivity Assays (Invitrogen) and total DNA was measured using a Qubit 4 Fluorometer (Invitrogen). A synthetic oligonucleotide standard (Integrated DNA Technologies) was used to calculate *16S rRNA* gene copies per μL using a ten-fold serial dilution.[22] *16S rRNA* gene real-time qPCR reactions were prepared using 5 μL of QIAGEN QuantiNova SYBR Green PCR Kit, a final concentration of 1μM of each primer, 1 μL of PCR grade water and 2 μL template DNA, to a final reaction volume of 10 μL. Prepared reactions were run on a Roche LightCycler 96 System for 15 minutes at 95°C, followed by 40 cycles of 95°C for 15 s and 58°C for 30 s. Amplification was followed by a dissociation curve (95°C for 10 s, 65°C for 60 s and 97°C for 1 s) to ensure the correct target sequence was being amplified. Each mosquito DNA extract was run in triplicate alongside standard curves and no template controls. PCR results were analyzed using the LightCycler 96 software (Roche Diagnostics).

Multilocus strain typing (MLST)

Wolbachia strains were characterized using the sequences of five conserved genes as molecular markers to genotype each strain.[47] PCR reactions and Sanger sequencing of PCR products were carried out as previously described.[18] Sequencing analysis was carried out in MEGAX[46] with consensus sequences used to perform nucleotide BLAST (NCBI) database queries, and for *Wolbachia* gene searches against the *Wolbachia* MLST database (https://pubmlst.org/wolbachia). Sanger sequencing traces from the *wsp* gene were also treated in the same way and analyzed alongside the MLST gene locus scheme, as an additional marker for strain typing. All *Wolbachia* gene sequence GenBank accession numbers are listed in Table S1.

Phylogenetic analysis

Alignments were constructed in MEGAX[46] by ClustalW to include relevant sequences highlighted through searches on the BLAST and *Wolbachia* MLST databases. Maximum Likelihood phylogenetic trees were constructed from Sanger sequences as follows. The evolutionary history was inferred by using the Maximum Likelihood method based on the Tamura-Nei model.[75] The tree with the highest log likelihood in each case is shown. The percentage of trees in which the associated taxa clustered together is shown next to the branches. Initial tree(s) for the heuristic search were obtained automatically by applying Neighbor-Join and BioNJ algorithms to a matrix of pairwise distances estimated using the Maximum Composite Likelihood (MCL) approach, and then selecting the topology with superior log likelihood value. The trees are drawn to scale, with branch lengths measured in the number of substitutions per site. Codon positions included were 1st+2nd+3rd+Noncoding. All positions containing gaps and missing data were eliminated. The phylogeny test was by Bootstrap method with 1000 replications. Evolutionary analyses were conducted in MEGAX.[46]

Microbiome analysis

The microbiomes of selected individual mosquitoes were analyzed using barcoded high-throughput amplicon sequencing of the bacterial *16S rRNA* gene (with library preparation and Illumina sequencing carried out commercially by Source Bioscience, Cambridge, UK). Sequencing of each extract was generated using universal *16S rRNA* V3-V4 region primers (FOR: CCTACGGGNGGCWGCAG, REV: GGACTACHVGGGTATCTAATCC)[76] using standard Illumina *16S rRNA* metagenomic sequencing library protocols with Nextera transposase adapters and IDT – Illumina Nextera Unique Dual Indexes. Amplicon PCRs were undertaken using a 2x KAPA HiFi HotStart ReadyMix PCR Kit with 12.5 ng of total DNA in 25 mL reactions. AMPure XP beads were used to purify the *16S* V3 and V4 amplicon followed by index barcoding using a KAPA HiFi HotStart ReadyMix PCR Kit. A final clean-up of the library using AMPure XP beads was undertaken prior to validation of the final library using the D1000 ScreenTape Assay on the Agilent TapeStation 4200 to check size distribution and the Qubit High Sensitivity Assay to measure the concentration. The samples were pooled and loaded at a concentration of 4pM onto a flow cell and sequenced on an Illumina MiSeq, with the MiSeq v3 (600 cycle) reagent kit. Libraries were sequenced using 250bp PE, with 20% PhiX. Microbiome bioinformatics analyses were carried out on demultiplexed reads using QIIME2 Core (q2cli) 2019.7 distribution.[24] Due to low sequencing yield, only single-end (R2) reads were used for analysis. Demultiplexed reads were imported and then primers were removed using the q2-cutadapt plugin.[48]

Quality plots were generated and visualized using the q2-demux summarize command to assess and select optimal quality filtering parameters including truncation length for any adaptor sequence removal. Quality filtering (p-trunc-len 227), Denoising and Chimera Removal was carried out using the q2-dada2 plugin[49] to group Amplicon Sequence Variants (ASVs) within the data. Taxonomic assignment of ASVs was carried out using the q2-feature-classifier plugin[50] (qiime feature-classifier classify-sklearn command)[77] with a pre-trained SILVA classifier (Naive Bayes classifier was pre-trained on the *16S rRNA* SILVA SSU v132 97% reference database,[51] with the V3-V4 primers, provided by Source BioScience). The taxonomic assignments were visualized using qiime taxa barplot to show relative taxonomic abundance across all individual samples (Figure S4). Samples were grouped by species using qiime feature-table filter-samples. Summary average taxonomic abundances for each group were generated using qiime feature-table group (p-mode mean-ceiling), and then visualized using the qiime taxa barplot command. *Wolbachia* % taxonomic abundance of total *16S* bacterial load box-and-whisker plots were generated in GraphPad Prism.

Fluorescent *in situ* hybridization (FISH)

Freshly dead adult female mosquitoes were fully submerged in 100% acetone after removal of all legs and wings. Whole mosquitoes were embedded in paraffin wax and sectioned at Liverpool Bio-Innovation Hub (University of Liverpool). The FISH protocol was conducted as previously reported.[78] Briefly, sections were deparaffinated with three 5-minute washes in 100% Xylene, one 5-minute wash in 100% EtOH and one 5-minute wash in 95% EtOH. Slides were then placed in 6% H_2O_2 and 80% EtOH for at least 4 days. Slides were washed with diH_2O and 50ng of Wol3_Red (/5ATTO590N/TCCTCTATCCTCTTTCAATC) and 50ng of Wol4_Red (GAGTTAGCCAGGACTTCTTC/3ATTO590N/) were added to 500 μL of hybridization buffer pre-heated to 37°C.[45] Buffer containing the probes was placed on the slide and slides were placed in a hybridization chamber overnight at 37°C. Slides were washed once in 1x saline sodium citrate (SSC) (10mM DTT) for 15 mins, twice in 1x SSC (10mM DTT) for 15 mins at 55°C, twice in 0.5x SSC (10mM DTT) for 15 mins at 55°C, and finally, once in 0.5x SSC (10mM DTT) for 15 mins. Slides were again washed with diH_2O and 2 μL of DAPI in 200 μL of 1x PBS was placed on the tissue for 8 minutes. Slides were washed with 1x PBS and slides were mounted with a drop of anti-fade. No-probe and competition controls were undertaken. We also included positive controls which were *Cx. quinquefasciatus and Ae. albopictus* mosquitoes that harbor natural strains of *Wolbachia*. Images were captured with a Revolve FL microscope (Echolab).

Genome sequencing

Genomic DNA individually extracted from adult female *An. gambiae* s.s. (n = 4), *An. demeilloni* (n = 3), *An. coluzzii* (n = 1) and *An. moucheti* (n = 1) was used to generate sequencing libraries using Illumina Nextera DNA Flex transposase mediated kits according to manufacturer's protocols. Libraries were sequenced on an Illumina NextSeq 550 system with paired-end reads with a length of 150 bp (400M reads per run). Raw pair-ended reads were trimmed for Illumina Nextera adaptor sequences using Trimmomatic.[52] Reads were also quality-trimmed with Trimmomatic to a minimum PHRED quality of 20 within a sliding window of 4, discarding reads that fell below a minimum length of 100 base-pairs. Subsequently, host mosquito reads were removed from the samples. As no reference genome exists for either *An. moucheti* or *An. demeilloni*, genome assemblies of *An. gambiae* s.s. (GenBank: GCA_000005575.2), *An. funestus* (GenBank: GCA_003951495.1), and *An. arabiensis* (GenBank: GCA_000349185.1) were downloaded from VectorBase (accessed 14/02/2020).[53]

The trimmed pair-ended reads were mapped against the genome of *An. gambiae* s.s. (GenBank: GCA_000005575.2) using the BWA aligner with default settings (version 0.7.17-r1188).[54] Unmapped reads were extracted from the alignment and remapped against the genome of *An. funestus* (GenBank: GCA_003951495.1), before remaining unmapped reads were extracted and remapped to the genome of *An. arabiensis* (GenBank: GCA_000349185.1). Only reads that remained after this sequential remapping to three different *Anopheles* mosquito genomes were taken forward for *de novo* genome assembly. *De novo* genome assembly was conducted using the program MEGAHit (version 1.2.9)[55] with default parameters, which utilizes succinct de-brujin graphs for resource-efficient assembly of contigs from metagenomic data. This generated two sets of contigs from the two different mosquito species that were then analyzed with MetaQUAST (version 5.0.2, 0bb1dd1b)[56] to identify microbial species present within the dataset. The closest *Wolbachia* genome of *Drosophila (D.) simulans* strain Noumea (*w*No)[79] was selected (NCBI accession number CP003883.1). The *w*No genome was used to create a BlastN database and all contigs generated by MEGAHit (version 1.2.9)[55] were searched against the *w*No genome to identify contigs that are of likely *Wolbachia* origin within the two *Anopheles* species. These identified contigs were scaffolded against the *w*No genome using the Mauve contig mover (snapshot 2015-02-13).[57,58]

Reads from the two mosquito datasets were remapped to their corresponding draft genome assembly with the BWA-MEM aligner (version 0.171-r1188)[54] using default settings and average read depth calculated for each contig using the program samtools depth.[59] Contigs that showed greater than one standard deviation from the average read depth were removed from the assembly. Subsequent to the removal of these contigs, the reads were remapped to the draft genome and subsequently used to improve the assembly using the program Pilon (version 1.23).[60] Pilon automatically detects the presence of single nucleotide variants, or insertions/deletion events introduced during the assembly process. This was repeated a total of three times until no further insertion/deletions were detected.

Genome annotation and comparisons to existing genomes and sequence data

Annotation of both *Wolbachia* genomes was performed using the program PROKKA (version 1.11)[61] using default settings. This annotation was used to check for genome completeness using CheckM (version 1.1.2)[62] and identification of *cif* genes. The program

CellPress
OPEN ACCESS

CheckM utilizes a set of 'marker' genes that are present as single copy, and prevalent at > 97% in bacterial genomes within particular phylogenetic lineages to assess completeness.[62] In addition to the two draft genomes assembled during this study, an additional 48 *Wolbachia* genomes available on the NCBI database were also analyzed for comparison (Table S7). CheckM identified all but one of the analyzed input genomes as part of the Rickettsiales lineage, which *Wolbachia* is a member of, that contained a total of 368 marker genes.

The draft genome sequences for both *Wolbachia* strains were used as input into the program FastANI (version 1.3)[63] along with a selection of 48 additional *Wolbachia* genomes (list included in Table S7). FastANI utilizes the Average Nucleotide Identity (ANI) metric to check where genomes of different organisms may cluster together and can be used to determine supergroup placement of *Wolbachia* strains based on the entire genome sequence (rather than a selection of only up to six genes). FastANI allows for fast, alignment-free calculation of ANI scores of whole genomes to determine whether particular genomes cluster well together in terms of sequence identity and can be used to infer placement of supergroups for *Wolbachia*.

The outputs of FastANI were plotted as a heatmap, using the gplot's heatmap.2 library.[64] Annotation of protein domains within cytoplasmic incompatibility factor genes was performed using the HHPred webserver (accessed 15/06/2020)[65] with default parameters. Stop-codons, or frameshift mutations were manually removed from putative pseudogenes, and the amino acid sequences queried individually against the following databases: SCOPe70 (ver. 2.07), Pfam-A (ver. 33.1), COG_KOG (ver. 1.0), SMART (ver. 6.0). Graphics depicting the *cif* genes were generated using the R package gggenes, part of ggplot2.[66] The two assembled genomes were compared against one-another, as well as the genome assembly for *Wolbachia* of *D. melanogaster* (*w*Mel) (GenBank: GCA_000008025.1), *D. simulans* strain Noumea (*w*No) (GenBank: GCA_000376585.1), and *Ae. albopictus* (*w*AlbB) (GenBank: GCA_004795415.1), using the program Blast Ring Image Generator (version 0.95)[67] with default analysis options.

Genome mapping comparison

Comparison of read coverage depth between *Wolbachia* of different hosts was performed to analyze the density of *Wolbachia* infection. From this, we expect that sequencing datasets from arthropods with no known *Wolbachia* endosymbiont will have few or no reads mapping against any *Wolbachia* genome, and vice versa with sequencing datasets from arthropods with a known *Wolbachia* endosymbiont. Sequencing datasets from *Glossina brevipalpis* come from an arthropod known to host a *Wolbachia* endosymbiont, but no corresponding *Wolbachia* genome is available. In this case, we still expected that there will still be a significant number of reads mapping to related *Wolbachia* genomes. Sequencing datasets utilized were downloaded from the European Nucleotide Archive, with the full list available in Table S7. Sequences were trimmed using Trimmomatic (version 0.39)[52] to a minimum PHRED quality of 15 within a sliding window of four, discarding reads that fell below a minimum length of 50 base-pairs. Reads were then mapped to the corresponding host genome with the BWA-MEM aligner (version 0.171-r1188).[48] The resultant BAM file was then used as input into BEDTools' genomeCoverageBed program (version 2.29.2)[68] with no additional options. From this, a 'genomeCoverageBed.outfile is generated which contains a summary of read depths for each nucleotide position in the BAM file. This was then used as input into custom awk scripts (as detailed below) to calculate the depth and breadth of genome coverage. Remaining unmapped reads were extracted, and separately mapped to a selection of different *Wolbachia* genomes with mean depth and percentage breadth of coverage again calculated using the genomeCoverageBed program. The percentage breadth of coverage, as well as the log10-transformed mean depth of coverage was plotted onto a heatmap using ggplot2.[66] Heatmap plots for genome coverage were generated by first concatenating all contigs of the individual genomes into two long FASTA files. Sequencing data was then remapped against this composite FASTA file, and depth of read coverage calculated in 10,000 nucleotide base-pair windows using Bedtools' makewindows and coverage commands (version 2.29.2).[68] This was log10-transformed using base R commands, and plotted as a heatmap using the R package ggplot2.[66]

For calculating read depth of coverage: awk -F "\t" '{sum+ = $2*$3} END{print FILENAME "\t" sum}' genomeCoverage Bed.outfile

For calculating read percentage breadth of coverage: awk -F "\t" '$1 = = "genome" && $2 = = "0" {print FILENAME "\t" $3}' genomeCoverageBed.outfile

QUANTIFICATION AND STATISTICAL ANALYSIS

Normalized qPCR *Wolbachia 16S* rRNA gene copies per µL were compared using unpaired and paired t tests in GraphPad Prism 7. Statistical comparisons using t tests are presented in the results section with the number of mosquitoes analyzed (n), the t statistic (t), degrees of freedom (df) and the calculated probability (p) value.

Current Biology, Volume *31*

Supplemental Information

Stable high-density and maternally inherited *Wolbachia* infections in *Anopheles moucheti* and *Anopheles demeilloni* mosquitoes

Thomas Walker, Shannon Quek, Claire L. Jeffries, Janvier Bandibabone, Vishaal Dhokiya, Roland Bamou, Mojca Kristan, Louisa A. Messenger, Alexandra Gidley, Emily A. Hornett, Enyia R. Anderson, Cintia Cansado-Utrilla, Shivanand Hegde, Chimanuka Bantuzeko, Jennifer C. Stevenson, Neil F. Lobo, Simon C. Wagstaff, Christophe Antonio Nkondjio, Seth R. Irish, Eva Heinz, and Grant L. Hughes

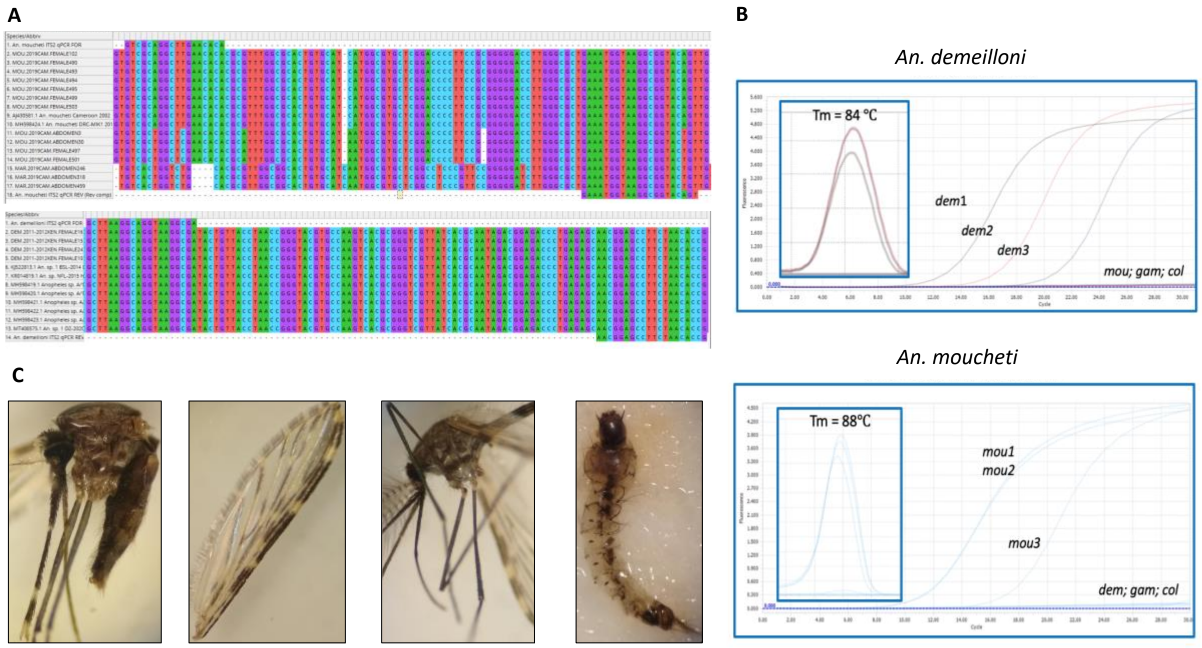

Figure S1: Mosquito identification using qPCR and morphological features. Related to Figure 1.
(A) Alignment of ITS2 sequences and location of species-specific primers.
(B) ITS2 species-specific qPCR fluorescence targeting *An. demeilloni* and *An. moucheti*. Inset = dissociation curves to ensure the correct target sequence was being amplified. dem = *An. demeilloni*, *mou* = *An. moucheti*, *gam* = *An. gambiae* s.s., *col* = *An. coluzzii*.
(C) Images of '*An. species* A'. From left to right: adult female, wing of adult female, adult male and larvae. Independent morphological identification by three individuals using keys confirmed this species is *An. demeilloni*.

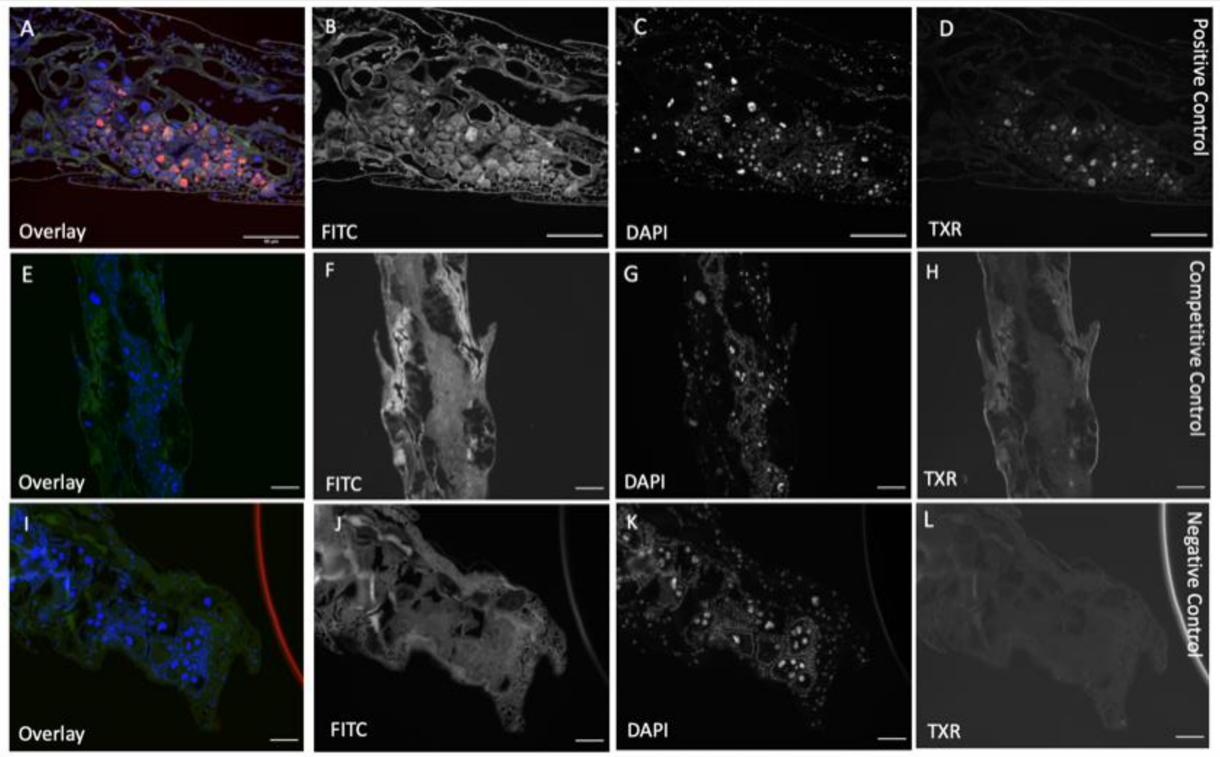

Figure S2: Controls for FISH. Related to Figure 2. Scale bars 90μM.
(A-D) *Wolbachia*-infected *Cx. quinquefasciatus* samples used as a positive control
(E-H) *An. moucheti* competitive control
(I-L) *An. moucheti* no probe control

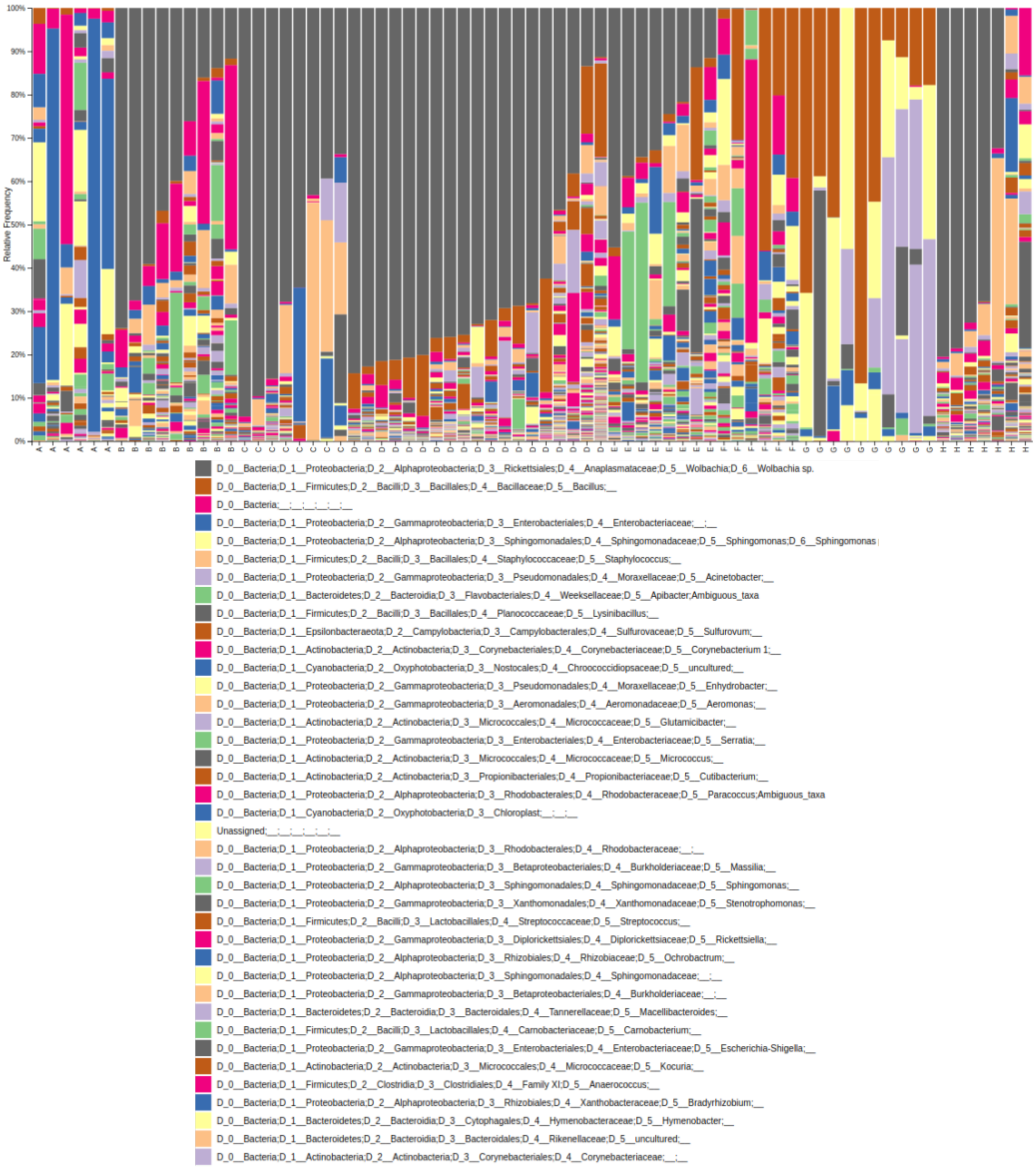

Group ID	Species (body part/generation)	Country (year)	*Wolbachia*+/total
A	*An. demeilloni* (whole/G0)	DRC (2015)	0/6
B	*An. demeilloni* (whole/G0)	DRC (2015)	9/9
C	*An. demeilloni* (whole/G0)	DRC (2019)	8/8
D	*An. moucheti* (abdomen/G0)	Cameroon (2019)	19/19
E	*An. moucheti* (head-thorax/G0)	Cameroon (2019)	8/8
F	*An. moucheti* (head-thorax/G0)	Cameroon (2019)	0/6
G	*An. moucheti* (whole/G0)	Cameroon (2019)	0/10
H	*An. moucheti* (whole/F1)	Cameroon (2019)	5/7

Figure S3: Microbiome relative taxonomic abundance barplots. Related to Figure 3. The relative taxonomic abundance barplots for each sample, as visualised using the qiime taxa barplot command within QIIME2. Sample groups with metadata are as detailed in the table and the legend details the level 7 classification of the 20 most abundant ASVs across all samples. Samples are arranged by group, then by descending % *Wolbachia*. The overwhelming dominance of *Wolbachia* within the microbiome of *An. demeilloni* and *An. moucheti* samples in the *Wolbachia* positive groups can be seen. In addition, the presence of high numbers of *Wolbachia* reads across different years for *An. demeilloni* (groups B and C), and in both the abdomen and head-thorax in *An. moucheti* (groups C and D) is shown. The maternal transmission of *Wolbachia* is demonstrated in the F1 *An. moucheti Wolbachia* positive samples within group H. The diversity of microbes present in these mosquitoes when *Wolbachia* is absent can also been seen (groups A, F, G and H).

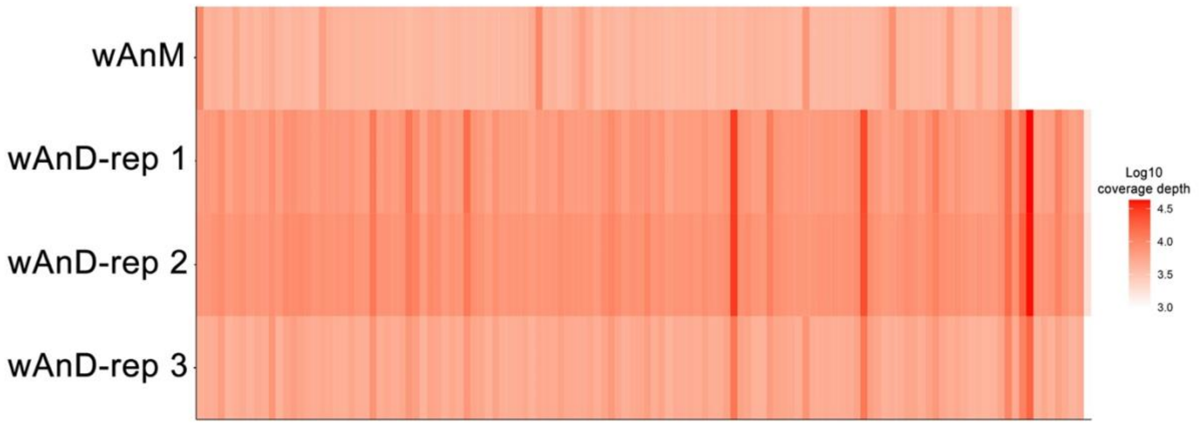

Figure S4. Heatmap representing depth of coverage for the assembled *w*AnM and *w*AnD genomes within 10kbp windows. Related to Figure 5.
Contigs for both genomes were first concatenated into one long assembly, before being separated into 10kbp-long windows. Sequencing data from individual samples were then mapped against the genome, and sequencing depth for each 10kbp window then calculated. Each row represents a single sample that has been aligned to one of the two genomes *w*AnM or *w*AnD, with intensity of red indicating the depth of sequencing as shown by the key to the right.

Sample ID	Location	Year	Species	Gene fragment	GenBank Accession Number
MOU.2015DRC.ABDOMEN7	Lwiro, DRC	2015	*An. moucheti moucheti*	Mosquito host COII	MW250655
MOU.2015DRC.ABDOMEN8	Lwiro, DRC	2015	*An. moucheti moucheti*	Mosquito host COII	MW250656
MOU.2019CAM.FEMALE30	Olama Village, CAM	2019	*An. moucheti moucheti*	Mosquito host COII	MW250657
MOU.2019CAM.FEMALE102	Olama Village, CAM	2019	*An. moucheti moucheti*	Mosquito host COII	MW250658
MOU.2019CAM.FEMALE490	Olama Village, CAM	2019	*An. moucheti moucheti*	Mosquito host COII	MW250659
MOU.2019CAM.FEMALE491	Olama Village, CAM	2019	*An. moucheti moucheti*	Mosquito host COII	MW250660
MOU.2019CAM.FEMALE493	Olama Village, CAM	2019	*An. moucheti moucheti*	Mosquito host COII	MW250661
MOU.2019CAM.FEMALE494	Olama Village, CAM	2019	*An. moucheti moucheti*	Mosquito host COII	MW250662
MOU.2019CAM.FEMALE495	Olama Village, CAM	2019	*An. moucheti moucheti*	Mosquito host COII	MW250663
MOU.2019CAM.FEMALE497	Olama Village, CAM	2019	*An. moucheti cf. moucheti*	Mosquito host COII	MW250664
MOU.2019CAM.FEMALE499	Olama Village, CAM	2019	*An. moucheti moucheti*	Mosquito host COII	MW250665
MOU.2019CAM.FEMALE500	Olama Village, CAM	2019	*An. moucheti moucheti*	Mosquito host COII	MW250666
MOU.2019CAM.FEMALE501	Olama Village, CAM	2019	*An. moucheti cf. moucheti*	Mosquito host COII	MW250667
MOU.2019CAM.FEMALE503	Olama Village, CAM	2019	*An. moucheti moucheti*	Mosquito host COII	MW250668
MOU.2019CAM.ABDOMEN1	Olama Village, CAM	2019	*An. moucheti moucheti*	Mosquito host COII	MW250669
MOU.2019CAM.ABDOMEN2	Olama Village, CAM	2019	*An. moucheti moucheti*	Mosquito host COII	MW250670
MOU.2019CAM.ABDOMEN3	Olama Village, CAM	2019	*An. moucheti cf. moucheti*	Mosquito host COII	MW250671
MOU.2019CAM.ABDOMEN6	Olama Village, CAM	2019	*An. moucheti moucheti*	Mosquito host COII	MW250672
MOU.2019CAM.ABDOMEN7	Olama Village, CAM	2019	*An. moucheti moucheti*	Mosquito host COII	MW250673
MOU.2019CAM.ABDOMEN9	Olama Village, CAM	2019	*An. moucheti moucheti*	Mosquito host COII	MW250674
MOU.2019CAM.ABDOMEN10	Olama Village, CAM	2019	*An. moucheti moucheti*	Mosquito host COII	MW250675
MOU.2019CAM.ABDOMEN11	Olama Village, CAM	2019	*An. moucheti moucheti*	Mosquito host COII	MW250676
MOU.2019CAM.ABDOMEN12	Olama Village, CAM	2019	*An. moucheti moucheti*	Mosquito host COII	MW250677
MOU.2019CAM.ABDOMEN13	Olama Village, CAM	2019	*An. moucheti moucheti*	Mosquito host COII	MW250678
MOU.2019CAM.ABDOMEN15	Olama Village, CAM	2019	*An. moucheti moucheti*	Mosquito host COII	MW250679
MOU.2019CAM.ABDOMEN17	Olama Village, CAM	2019	*An. moucheti moucheti*	Mosquito host COII	MW250680
MOU.2019CAM.ABDOMEN18	Olama Village, CAM	2019	*An. moucheti moucheti*	Mosquito host COII	MW250681
MOU.2019CAM.ABDOMEN19	Olama Village, CAM	2019	*An. moucheti moucheti*	Mosquito host COII	MW250682
MOU.2019CAM.ABDOMEN20	Olama Village, CAM	2019	*An. moucheti moucheti*	Mosquito host COII	MW250683
MOU.2019CAM.ABDOMEN21	Olama Village, CAM	2019	*An. moucheti moucheti*	Mosquito host COII	MW250684
MOU.2019CAM.ABDOMEN30	Olama Village, CAM	2019	*An. moucheti cf. moucheti*	Mosquito host COII	MW250685
MAR.2019CAM.ABDOMEN246	Olama Village, CAM	2019	*An. marshallii*	Mosquito host COII	MW250686
MAR.2019CAM.ABDOMEN318	Olama Village, CAM	2019	*An. marshallii*	Mosquito host COII	MW250687
MAR.2019CAM.ABDOMEN459	Olama Village, CAM	2019	*An. marshallii*	Mosquito host COII	MW250688
DEM.2019DRC.FEMALE3	Lwiro, DRC	2019	*An. demeilloni*	Mosquito host COII	MW250689
DEM.2011-2012KEN.FEMALE16	Western Highlands, KEN	2011-2012	*An. demeilloni*	Mosquito host COII	MW250690
DEM.2011-2012KEN.FEMALE15	Western Highlands, KEN	2011-2012	*An. demeilloni*	Mosquito host COII	MW250691
DEM.2011-2012KEN.FEMALE24	Western Highlands, KEN	2011-2012	*An. demeilloni*	Mosquito host COII	MW250692
DEM.2011-2012KEN.FEMALE10	Western Highlands, KEN	2011-2012	*An. demeilloni*	Mosquito host COII	MW250693
MOU.2019CAM.FEMALE102	Olama Village, CAM	2019	*An. moucheti moucheti*	Mosquito host ITS2	MW257124
MOU.2019CAM.FEMALE490	Olama Village, CAM	2019	*An. moucheti moucheti*	Mosquito host ITS2	MW257125
MOU.2019CAM.FEMALE491	Olama Village, CAM	2019	*An. moucheti moucheti*	Mosquito host ITS2	MW257126
MOU.2019CAM.FEMALE493	Olama Village, CAM	2019	*An. moucheti moucheti*	Mosquito host ITS2	MW257127
MOU.2019CAM.FEMALE494	Olama Village, CAM	2019	*An. moucheti moucheti*	Mosquito host ITS2	MW257128
MOU.2019CAM.FEMALE495	Olama Village, CAM	2019	*An. moucheti moucheti*	Mosquito host ITS2	MW257129
MOU.2019CAM.FEMALE499	Olama Village, CAM	2019	*An. moucheti moucheti*	Mosquito host ITS2	MW257130
MOU.2019CAM.FEMALE500	Olama Village, CAM	2019	*An. moucheti moucheti*	Mosquito host ITS2	MW257131
MOU.2019CAM.FEMALE503	Olama Village, CAM	2019	*An. moucheti moucheti*	Mosquito host ITS2	MW257132
MOU.2019CAM.FEMALE497	Olama Village, CAM	2019	*An. moucheti cf. moucheti*	Mosquito host ITS2	MW257133
MOU.2019CAM.FEMALE501	Olama Village, CAM	2019	*An. moucheti cf. moucheti*	Mosquito host ITS2	MW257134
MOU.2019CAM.ABDOMEN3	Olama Village, CAM	2019	*An. moucheti cf. moucheti*	Mosquito host ITS2	MW257135
MOU.2019CAM.ABDOMEN30	Olama Village, CAM	2019	*An. moucheti cf. moucheti*	Mosquito host ITS2	MW257136
MAR.2019CAM.ABDOMEN246	Olama Village, CAM	2019	*An. marshallii*	Mosquito host ITS2	MW257137
MAR.2019CAM.ABDOMEN318	Olama Village, CAM	2019	*An. marshallii*	Mosquito host ITS2	MW257138
MAR.2019CAM.ABDOMEN459	Olama Village, CAM	2019	*An. marshallii*	Mosquito host ITS2	MW257139
MOU.2015DRC.ABDOMEN1	Lwiro, DRC	2015	*An. moucheti moucheti*	Mosquito host ITS2	MW257140
MOU.2015DRC.ABDOMEN2	Lwiro, DRC	2015	*An. moucheti moucheti*	Mosquito host ITS2	MW257141
MOU.2015DRC.ABDOMEN3	Lwiro, DRC	2015	*An. moucheti moucheti*	Mosquito host ITS2	MW257142

MOU.2015DRC.ABDOMEN4	Lwiro, DRC	2015	*An. moucheti moucheti*	Mosquito host ITS2	MW257143
MOU.2015DRC.ABDOMEN5	Lwiro, DRC	2015	*An. moucheti moucheti*	Mosquito host ITS2	MW257144
MOU.2015DRC.ABDOMEN6	Lwiro, DRC	2015	*An. moucheti moucheti*	Mosquito host ITS2	MW257145
MOU.2015DRC.ABDOMEN7	Lwiro, DRC	2015	*An. moucheti moucheti*	Mosquito host ITS2	MW257146
MOU.2015DRC.ABDOMEN8	Lwiro, DRC	2015	*An. moucheti moucheti*	Mosquito host ITS2	MW257147
2011-2012.KEN.FEMALE16	Western Highlands, KEN	2011-2012	*An. demeilloni*	Mosquito host ITS2	MW257148
2011-2012.KEN.FEMALE15	Western Highlands, KEN	2011-2012	*An. demeilloni*	Mosquito host ITS2	MW257149
2011-2012.KEN.FEMALE24	Western Highlands, KEN	2011-2012	*An. demeilloni*	Mosquito host ITS2	MW257150
2011-2012.KEN.FEMALE10	Western Highlands, KEN	2011-2012	*An. demeilloni*	Mosquito host ITS2	MW257151
MOU.2015DRC.ABDOMEN3 wsp(1)	Lwiro, DRC	2015	*An. moucheti moucheti*	Wolbachia wsp	MW250694
MOU.2019CAM.ABDOMEN1	Olama Village, CAM	2019	*An. moucheti moucheti*	Wolbachia wsp	MW250695
MOU.2019CAM.ABDOMEN2	Olama Village, CAM	2019	*An. moucheti moucheti*	Wolbachia wsp	MW250696
MOU.2019CAM.ABDOMEN3 wsp(2)	Olama Village, CAM	2019	*An. moucheti cf. moucheti*	Wolbachia wsp	MW250697
MOU.2019CAM.ABDOMEN6 wsp(1)	Olama Village, CAM	2019	*An. moucheti moucheti*	Wolbachia wsp	MW250698
MOU.2019CAM.ABDOMEN7	Olama Village, CAM	2019	*An. moucheti moucheti*	Wolbachia wsp	MW250699
MOU.2019CAM.ABDOMEN8	Olama Village, CAM	2019	*An. moucheti*	Wolbachia wsp	MW250700
MOU.2019CAM.ABDOMEN9	Olama Village, CAM	2019	*An. moucheti moucheti*	Wolbachia wsp	MW250701
MOU.2019CAM.ABDOMEN10	Olama Village, CAM	2019	*An. moucheti moucheti*	Wolbachia wsp	MW250702
MOU.2019CAM.ABDOMEN11	Olama Village, CAM	2019	*An. moucheti moucheti*	Wolbachia wsp	MW250703
MOU.2019CAM.ABDOMEN12	Olama Village, CAM	2019	*An. moucheti moucheti*	Wolbachia wsp	MW250704
MOU.2019CAM.ABDOMEN13	Olama Village, CAM	2019	*An. moucheti moucheti*	Wolbachia wsp	MW250705
MOU.2019CAM.ABDOMEN15	Olama Village, CAM	2019	*An. moucheti moucheti*	Wolbachia wsp	MW250706
MOU.2019CAM.ABDOMEN16	Olama Village, CAM	2019	*An. moucheti*	Wolbachia wsp	MW250707
MOU.2019CAM.ABDOMEN17	Olama Village, CAM	2019	*An. moucheti moucheti*	Wolbachia wsp	MW250708
MOU.2019CAM.ABDOMEN18	Olama Village, CAM	2019	*An. moucheti moucheti*	Wolbachia wsp	MW250709
MOU.2019CAM.ABDOMEN19	Olama Village, CAM	2019	*An. moucheti moucheti*	Wolbachia wsp	MW250710
MOU.2019CAM.ABDOMEN20	Olama Village, CAM	2019	*An. moucheti moucheti*	Wolbachia wsp	MW250711
MOU.2019CAM.ABDOMEN30 wsp(2)	Olama Village, CAM	2019	*An. moucheti cf. moucheti*	Wolbachia wsp	MW250712
MOU.2019CAM.FEMALE102 wsp(1)	Olama Village, CAM	2019	*An. moucheti moucheti*	Wolbachia wsp	MW250713
2011-2012.KEN.FEMALE16	Western Highlands, KEN	2011-2012	*An. demeilloni*	Wolbachia wsp	MW250714
2011-2012.KEN.FEMALE15	Western Highlands, KEN	2011-2012	*An. demeilloni*	Wolbachia wsp	MW250715
2011-2012.KEN.FEMALE24	Western Highlands, KEN	2011-2012	*An. demeilloni*	Wolbachia wsp	MW250716
MOU.2019CAM.ABDOMEN3	Olama Village, CAM	2019	*An. moucheti cf. moucheti*	Wolbachia coxA	MW250717
MOU.2019CAM.ABDOMEN30	Olama Village, CAM	2019	*An. moucheti cf. moucheti*	Wolbachia coxA	MW250718
MOU.2019CAM.FEMALE102	Olama Village, CAM	2019	*An. moucheti moucheti*	Wolbachia coxA	MW250719
MOU.2015DRC.ABDOMEN3	Lwiro, DRC	2015	*An. moucheti moucheti*	Wolbachia coxA	MW250720
MOU.2015DRC.ABDOMEN4	Lwiro, DRC	2015	*An. moucheti moucheti*	Wolbachia coxA	MW250721
MOU.2015DRC.ABDOMEN7	Lwiro, DRC	2015	*An. moucheti moucheti*	Wolbachia coxA	MW250722
MOU.2015DRC.ABDOMEN8	Lwiro, DRC	2015	*An. moucheti moucheti*	Wolbachia coxA	MW250723
2011-2012.KEN.FEMALE16 (coxA-1)	Western Highlands, KEN	2011-2012	*An. demeilloni*	Wolbachia coxA	MW250724
2011-2012.KEN.FEMALE15 (coxA-1)	Western Highlands, KEN	2011-2012	*An. demeilloni*	Wolbachia coxA	MW250725
2011-2012.KEN.FEMALE15 (coxA-2)	Western Highlands, KEN	2011-2012	*An. demeilloni*	Wolbachia coxA	MW250726
2011-2012.KEN.FEMALE24 (coxA-1)	Western Highlands, KEN	2011-2012	*An. demeilloni*	Wolbachia coxA	MW250727
MOU.2019CAM.ABDOMEN3	Olama Village, CAM	2019	*An. moucheti cf. moucheti*	Wolbachia gatB	MW250728
MOU.2019CAM.ABDOMEN30	Olama Village, CAM	2019	*An. moucheti cf. moucheti*	Wolbachia gatB	MW250729
MOU.2019CAM.FEMALE102	Olama Village, CAM	2019	*An. moucheti moucheti*	Wolbachia gatB	MW250730
MOU.2015DRC.ABDOMEN3	Lwiro, DRC	2015	*An. moucheti moucheti*	Wolbachia gatB	MW250731
MOU.2015DRC.ABDOMEN4	Lwiro, DRC	2015	*An. moucheti moucheti*	Wolbachia gatB	MW250732
MOU.2015DRC.ABDOMEN7	Lwiro, DRC	2015	*An. moucheti moucheti*	Wolbachia gatB	MW250733
MOU.2015DRC.ABDOMEN8	Lwiro, DRC	2015	*An. moucheti moucheti*	Wolbachia gatB	MW250734
2011-2012.KEN.FEMALE16	Western Highlands, KEN	2011-2012	*An. demeilloni*	Wolbachia gatB	MW250735
2011-2012.KEN.FEMALE15	Western Highlands, KEN	2011-2012	*An. demeilloni*	Wolbachia gatB	MW250736
2011-2012.KEN.FEMALE24	Western Highlands, KEN	2011-2012	*An. demeilloni*	Wolbachia gatB	MW250737
MOU.2019CAM.ABDOMEN3	Olama Village, CAM	2019	*An. moucheti cf. moucheti*	Wolbachia ftsZ	MW250738
MOU.2019CAM.ABDOMEN30	Olama Village, CAM	2019	*An. moucheti cf. moucheti*	Wolbachia ftsZ	MW250739
MOU.2019CAM.FEMALE102	Olama Village, CAM	2019	*An. moucheti moucheti*	Wolbachia ftsZ	MW250740

MOU.2015DRC.ABDOMEN3	Lwiro, DRC	2015	*An. moucheti moucheti*	Wolbachia ftsZ	MW250741
MOU.2015DRC.ABDOMEN4	Lwiro, DRC	2015	*An. moucheti moucheti*	Wolbachia ftsZ	MW250742
MOU.2015DRC.ABDOMEN7	Lwiro, DRC	2015	*An. moucheti moucheti*	Wolbachia ftsZ	MW250743
MOU.2015DRC.ABDOMEN8	Lwiro, DRC	2015	*An. moucheti moucheti*	Wolbachia ftsZ	MW250744
2011-2012.KEN.FEMALE16	Western Highlands, KEN	2011-2012	*An. demeilloni*	Wolbachia ftsZ	MW250745
2011-2012.KEN.FEMALE15	Western Highlands, KEN	2011-2012	*An. demeilloni*	Wolbachia ftsZ	MW250746
2011-2012.KEN.FEMALE24	Western Highlands, KEN	2011-2012	*An. demeilloni*	Wolbachia ftsZ	MW250747
MOU.2019CAM.ABDOMEN3	Olama Village, CAM	2019	*An. moucheti cf. moucheti*	Wolbachia fpbA	MW250748
MOU.2019CAM.ABDOMEN30	Olama Village, CAM	2019	*An. moucheti cf. moucheti*	Wolbachia fpbA	MW250749
MOU.2019CAM.FEMALE102	Olama Village, CAM	2019	*An. moucheti moucheti*	Wolbachia fpbA	MW250750
MOU.2015DRC.ABDOMEN3	Lwiro, DRC	2015	*An. moucheti moucheti*	Wolbachia fpbA	MW250751
MOU.2015DRC.ABDOMEN4	Lwiro, DRC	2015	*An. moucheti moucheti*	Wolbachia fpbA	MW250752
MOU.2015DRC.ABDOMEN7	Lwiro, DRC	2015	*An. moucheti moucheti*	Wolbachia fpbA	MW250753
MOU.2015DRC.ABDOMEN8	Lwiro, DRC	2015	*An. moucheti moucheti*	Wolbachia fpbA	MW250754
2011-2012.KEN.FEMALE16	Western Highlands, KEN	2011-2012	*An. demeilloni*	Wolbachia fpbA	MW250755
2011-2012.KEN.FEMALE15	Western Highlands, KEN	2011-2012	*An. demeilloni*	Wolbachia fpbA	MW250756
2011-2012.KEN.FEMALE24	Western Highlands, KEN	2011-2012	*An. demeilloni*	Wolbachia fpbA	MW250757
MOU.2019CAM.ABDOMEN3	Olama Village, CAM	2019	*An. moucheti cf. moucheti*	Wolbachia hcpA	MW250758
MOU.2019CAM.ABDOMEN30	Olama Village, CAM	2019	*An. moucheti cf. moucheti*	Wolbachia hcpA	MW250759
MOU.2019CAM.FEMALE102	Olama Village, CAM	2019	*An. moucheti moucheti*	Wolbachia hcpA	MW250760
MOU.2015DRC.ABDOMEN3	Lwiro, DRC	2015	*An. moucheti moucheti*	Wolbachia hcpA	MW250761
MOU.2015DRC.ABDOMEN4	Lwiro, DRC	2015	*An. moucheti moucheti*	Wolbachia hcpA	MW250762
MOU.2015DRC.ABDOMEN7	Lwiro, DRC	2015	*An. moucheti moucheti*	Wolbachia hcpA	MW250763
MOU.2015DRC.ABDOMEN8	Lwiro, DRC	2015	*An. moucheti moucheti*	Wolbachia hcpA	MW250764
2011-2012.KEN.FEMALE16	Western Highlands, KEN	2011-2012	*An. demeilloni*	Wolbachia hcpA	MW250765
2011-2012.KEN.FEMALE15	Western Highlands, KEN	2011-2012	*An. demeilloni*	Wolbachia hcpA	MW250766
2011-2012.KEN.FEMALE24	Western Highlands, KEN	2011-2012	*An. demeilloni*	Wolbachia hcpA	MW250767

Table S1: Additional Sanger sequencing sample details for *w*AnM-infected *An. moucheti* and *w*AnD-infected *An. demeilloni* with their associated GenBank accession numbers. Related to STAR Methods.
The location and year of collection, sample codes and the sequenced gene fragment is shown in addition to the GenBank accession number.

species	collection year	collection location	generation	mosquito body part/life cycle stage	Individual extracts analysed	% individuals *Wolbachia+*	mean *16S rRNA* gene copies /ng DNA
An. moucheti	2019	Olama, CAM	G0 (wild)	female whole body	520	44.2% (238/520)	1.48E+05
An. moucheti	2019	Olama, CAM	G0 (wild)	female abdomen	566	66.6% (377/566)	3.36E+05
An. moucheti	2019	Olama, CAM	G0 (wild)	female head-thorax	566	17.5% (99/566)	5.38E+04
An. moucheti	2019	Olama, CAM	F1	larvae	56	23.2% (13/56)	7.70E+03
An. moucheti	2019	Olama, CAM	F1	pupae	8	25.0% (2/8)	7.80E+03
An. moucheti	2019	Olama, CAM	F1	female whole body	7	85.7% (6/7)	2.36E+05
An. moucheti	2019	Olama, CAM	F1	male whole body	5	80.% (4/5)	1.12E+04
An. moucheti	2015	Lwiro, DRC	G0 (wild)	female abdomen	8	75.0% (6/8)	9.05E+03
An. demeilloni	2015	Lwiro, DRC	G0 (wild)	female whole body	178	89.3% (159/178)	6.67E+03
An. demeilloni	2019	Lwiro, DRC	G0 (wild)	female whole body	8	100.0% (8/8)	8.19E+05
An. demeilloni	2019	Lwiro, DRC	F1	eggs (pooled)	1*	100.0% (1/1)	6.99E+04
An. demeilloni	2019	Lwiro, DRC	F1	larvae	11	72.7% (8/11)	3.79E+03
An. demeilloni	2019	Lwiro, DRC	F1	pupae	9	100.0% (9/9)	1.58E+03
An. demeilloni	2019	Lwiro, DRC	F1	male whole body	10	70% (7/10)	2.93E+03
An. demeilloni	2019	Lwiro, DRC	F1	female abdomen	12	91.7% (11/12)	3.55E+04
An. demeilloni	2019	Lwiro, DRC	F1	female head-thorax	13	0.0% (0/13)	0.00E+00
An. demeilloni	2019	Lwiro, DRC	F2	female abdomen	11	90.9% (10/11)	5.76E+05
An. demeilloni	2019	Lwiro, DRC	F2	female head-thorax	11	18.2% (2/11)	5.91E+03
An. demeilloni	2011-2012	Western Highlands, KEN	G0 (wild)	female whole body	302	38.7% (117/302)	2.04E+05

Table S2: *Wolbachia* density of the *w*AnM and *wAnD* strains. Related to Figure 1. Mean *Wolbachia 16S rRNA* gene copies/ng DNA for *Wolbachia*-infected mosquito DNA extracts. *approximately 200 eggs were pooled prior to extraction. CAM = Cameroon, DRC = Democratic Republic of Congo, KEN = Kenya.

Mosquito species	Collection	strain	*WSP* typing allele numbers				
			wsp	HVR1	HVR2	HVR3	HVR4
An. moucheti	Olama, Cameroon	*w*AnM - VAR1	736	10	**296**	10	13
An. moucheti	Olama, Cameroon	*w*AnM - VAR2	**CM 322 (1 diff)**	10	**295**	10	13
An. demeilloni	Lwiro, DRC	*w*AnD	728	254	288	284	23
An. demeilloni	Western Highlands, Kenya	*w*AnD	728	254	288	284	23

Table S3: *w*AnM and *w*AnD *Wolbachia* strain *WSP* typing. Related to Figure 4.
The *wsp* sequence for *w*AnM-VAR2 had 1 nucleotide difference to allele number 322 (CM = closest match) and 3 differences to *w*AnM-VAR1 in hypervariable region (HVR) 2.

Average X mapping coverage	*Wolbachia* genomes 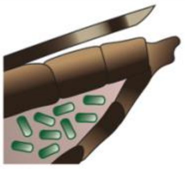		Mosquito genomes 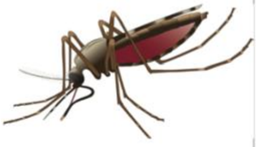	
	min	max	min	max
Unknown *Wolbachia* infection	0.01	2.83	6.29	149.78
An. gambiae (Burkina Faso)[○]	0.03	0.08	149.78	
An. gambiae (DRC)[◇]	0.06	0.11	62.56	
An. coluzzii (Ghana)	0.01	0.03	18.85	
Known *Wolbachia* infection	0.26	148.55	8.19	107.13
An. moucheti	9.40	62.07	34.50	
An. demeilloni	14.96	74.77	22.80	

Table S4: **Average mapping coverage of *Wolbachia* and host mosquito genomes. Related to Figure 5.** Average mapping coverage of *Wolbachia* and host mosquito genomes. The average minimum and maximum coverage are shown comparing *Anopheles* species and arthropods with or without a known *Wolbachia* strain. Due to no genome being available for *An. demeilloni* and *An. moucheti*, the genome of *An. funestus* was used for comparison (see methods).

**Strain identifier**	**Supergroup**	**Genome size**	**GC %**	**Contigs**	**Predicted genes**
*w*AnM	B	1,133,840	33.59%	179	1,164
*w*AnD	B	1,232,500	33.58%	64	1,314

Table S5. General characteristics of the *w*AnD and *w*AnM genomes. Related to Figure 5.

Accession	Short name	Host name	Publish date
GCA_000008025.1	wDmel	Drosophila melanogaster	16/01/2002
GCA_000073005.1	wPip	Culex quinquefasciatus Pel	13/06/2008
GCA_000376585.1	wNo	Drosophila simulans strain No	22/04/2013
GCA_000376605.1	wHa	Drosophila simulans strain Ha	22/04/2013
GCA_000475015.1	wMelPop	Drosophila melanogaster strain popcorn	21/10/2013
GCA_000530755.1	wOv	Onchocerca volvulus	24/01/2014
GCA_000953315.1	wAu	Drosophila simulans strain Au	15/10/2014
GCA_001648015.1	wDacB	Dactylopus coccus supergroup B	19/05/2016
GCA_001648025.1	wDacA	Dactylopus coccus supergroup A	19/05/2016
GCA_001758565.1	wInc_Cu	Drosophila incompta	11/10/2016
GCA_001758585.1	wInc_SM	Drosophila incompta	11/10/2016
GCA_001931755.2	wFcan	Folsoma candida	25/06/2018
GCA_002204235.2	wWb	Wuchereria bancrofti	07/07/2017
GCA_002300525.1	wSpc	Drosophila subpulchrella	13/09/2017
GCA_003344345.1	wCon	Cylisticus convexus	27/07/2018
GCA_003704235.1	wFex	Formica execta	31/10/2018
GCA_003999585.1	wBtab	Bemisia tabaci	08/01/2019
GCA_004795415.1	wAlbB	Aedes albopictus	15/04/2019
GCA_004795935.1	wBm	Brugia malayi	30/08/2019
GCA_004795975.1	wMau	Drosophila mauritania	15/04/2019
GCA_007115015.1	wStri	Laodelphax striatellus	18/07/2019
GCF_000174095.1	wUni	Muscidufax uniraptor	24/03/2009
GCF_000204545.1	wNvit	Nasonia vitripennis	22/04/2011
GCF_000306885.1	wOo	Onchocerca ochengi	30/07/2012
GCF_000331595.1	wDcit	Diaphoria citri	15/01/2013
GCF_000333795.1	wDsuzu	Drosophila suzukii	11/01/2013
GCF_000689175.1	wGmm	Glossina morsitans morsitans	09/05/2014
GCF_000723225.2	wPip_Mol	Culex molestus	20/04/2015
GCF_000829315.1	wClec	Cimex lectularius	10/06/2014
GCF_001027565.1	wAvul	Armadillidium vulgare	18/06/2015
GCF_001266585.1	wOpbru	Operophtera brumata	11/08/2015
GCF_001439985.1	wTPRE	Trichogramma pretiosum	07/01/2016
GCF_001675695.1	wNfla	Nomada flava	23/06/2016
GCF_001675715.1	wNleu	Nomada leucophthalma	23/06/2016
GCF_001675775.1	wNpa	Nomada panzeri	23/06/2016
GCF_001675785.1	wNferru	Nomada ferruginata	23/06/2016
GCF_005862095.1	wSan	Drosophila santomea	27/05/2019
GCF_005862115.1	wYak	Drosophila yakuba	27/05/2019
GCF_005862135.1	wTei	Drosophila teissieri	27/05/2019
GCF_006334525.1	wGBW	Leptopilina clavipes	12/06/2019
GCF_006542295.1	wCauA	Carposina sasakii	02/07/2019
GCF_007115045.1	wLug	Nilaparvata lugens	18/07/2019
GCF_008033215.1	wDana	Drosophila ananassae	20/08/2019
GCF_008245065.1	wMeg	Chrysoma megacephala	03/09/2019
GCF_009012935.1	wOneA1	Nasonia oneida	11/10/2019
wLs 2.0, http://nematodes.org/genomes/litomosoides_sigmodontis/	wLs	Litomosoides sigmodontis	06/08/2012
wDi, http://nematodes.org/genomes/dirofilaria_immitis/	wDi	Dirofilaria immitis	06/08/2012
-	wAnM_draft_genome	Anopheles moucheti	-
-	wAnD_draft_genome	Anopheles species A	-
Supplementary material, DOI 10.7717/peerj.5486	wAnsA_Pascar_genome	Anopheles species A	03/09/2018

Table S7: *Wolbachia* genomes used for comparison. **Related to STAR Methods.**
Existing *Wolbachia* genomes used in this study for comparison against the assembled genomes.

**Gene target and oligonucleotide primer sequences**	**Reference**
Mosquito ITS2:<br>5’-TGTGAACTTGCAGGACACAT-3’; 5’-TATGCTTAAATTYAGGGGGT-3’	S1
Mosquito *COII*:<br>5'-TCTAATATGGGAGATTAGTGC-3'; 5'-ACTTGCTTTCAGTCATCTAATG-3'	S2
Wolbachia wsp:<br>5’-TGGTCCAATAAGTGATGAAGAAAC-3’; 5’-AAAAATTAAACGCTACTCCA-3’	S3
Wolbachia 16S rRNA:<br>5’-CATACCTATTCGAAGGGATA-3’; 5’-AGCTTCGAGTGAAACCAATTC-3	S4
Wolbachia coxA:<br>5’-TTGGRGCRATYAACTTTATAG-3’; 5’-CTAAAGACTTTKACRCCAGT-3’	S5
Wolbachia ftsZ:<br>5’-ATYATGGARCATATAAARGATAG-3’; 5’-TCRAGYAATGGATTRGATAT-3’	S5
Wolbachia hcpA:<br>5’-GAAATARCAGTTGCTGCAAA-3’; 5’-GAAAGTYRAGCAAGYTCTG-3’	S5
Wolbachia fbpA:<br>5’-GCTGCTCCRCTTGGYWTGAT-3’; 5’-CCRCCAGARAAAAYYACTATTC-3	S5
Wolbachia gatB:<br>5’-GAKTTAAAYCGYGCAGGBGTT-3’; 5’-TGGYAAYTCRGGYAAAGATGA-3’	S5
Wolbachia 16S rRNA (qPCR):<br>5′-CATACCTATTCGAAGGGATAG-3′; 5′-TTGCGGGACTTAACCCAACA-3′	S6
An. moucheti ITS2:<br>5’-GTCGCAGGCTTGAACACA-3’; 5’-ACTGTACCGCCTTACCATTTC-3’	In this study
An. demeilloni ITS2<br>5’-GCTTAAGGCAGGTAAGGCGA-3’; 5’-CGGTGTTAGAAGGCTCCGTT-3’	In this study
Bacterial *16S rRNA* V3-V4:<br>5’-CCTACGGGNGGCWGCAG-3’; 5’-GGACTACHVGGGTATCTAATCC-3’	S7

Table S10: Primers used in this study. Related to STAR Methods.

**Species**	**number collected**
An. nili	1
An. paludis	26
An. gambiae s.l.	32
An. ziemanni	451
An. marshallii	855
An. moucheti s.l.	2652
Total	**4017**

Table S11. *Anopheles* species collected from Olama Village, Cameroon using human landing catches in 2019. Related to STAR Methods.

Supplemental References

S1. Beebe N.W., Saul A. Discrimination of all members of the Anopheles punctulatus complex by polymerase chain reaction--restriction fragment length polymorphism analysis. Am J Trop Med Hyg. 1995;**53**(5):478-81.

S2. Ndo C., Antonio-Nkondjio C., Cohuet A., Ayala D., Kengne P., Morlais I., Awono-Ambene P.H., Couret D., Ngassam P., Fontenille D., et al. Population genetic structure of the malaria vector Anopheles nili in sub-Saharan Africa. Malar J. 2010;**9**:161.

S3. Zhou W., Rousset F., O'Neil S. Phylogeny and PCR-based classification of Wolbachia strains using wsp gene sequences. Proc Biol Sci. 1998;**265**(1395):509-15.

S4. Werren J.H., Windsor D.M. Wolbachia infection frequencies in insects: evidence of a global equilibrium? Proc Biol Sci. 2000;**267**(1450):1277-85.

S5. Baldo L., Dunning Hotopp J.C., Jolley K.A., Bordenstein S.R., Biber S.A., Choudhury R.R., Hayashi C., Maiden M.C., Tettelin H., Werren J.H. Multilocus sequence typing system for the endosymbiont Wolbachia pipientis. Appl Environ Microbiol. 2006;**72**(11):7098-110.

S6. Gomes F.M., Hixson B.L., Tyner M.D.W., Ramirez J.L., Canepa G.E., Alves E.S.T.L., Molina-Cruz A., Keita M., Kane F., Traore B., et al. Effect of naturally occurring Wolbachia in Anopheles gambiae s.l. mosquitoes from Mali on Plasmodium falciparum malaria transmission. Proc Natl Acad Sci U S A. 2017;**114**(47):12566-71.

S7. Klindworth A., Pruesse E., Schweer T., Peplies J., Quast C., Horn M., Glockner F.O. Evaluation of general 16S ribosomal RNA gene PCR primers for classical and next-generation sequencing-based diversity studies. Nucleic Acids Res. 2013;**41**(1):e1.
